# Multifunctional Medical Grade Resin with Enhanced Mechanical and Antibacterial Properties: The Effect of Copper Nano-Inclusions in Vat Polymerization (VPP) Additive Manufacturing

**DOI:** 10.3390/jfb13040258

**Published:** 2022-11-21

**Authors:** Nectarios Vidakis, Markos Petousis, Vassilis M. Papadakis, Nikolaos Mountakis

**Affiliations:** 1 Department of Mechanical Engineering, Hellenic Mediterranean University, 71410 Heraklion, Greece; 2 Institute of Molecular Biology and Biotechnology, Foundation for Research and Technology—Hellas, 71110 Heraklion, Greece

**Keywords:** copper (Cu), vat photopolymerization, resin, additive manufacturing (AM), *Escherichia coli (E. coli)*, *Staphylococcus aureus (S. aureus)*, medical grade, amicrobial

## Abstract

Vat photopolymerization (VPP) is an additive manufacturing process commonly used in medical applications. This work aims, for the first time in the literature, to extend and enhance the performance of a commercial medical-grade resin for the VPP process, with the development of nanocomposites, using Copper (Cu) nanoparticles as the additive at two different concentrations. The addition of the Cu nanoparticles was expected to enhance the mechanical properties of the resin and to enable biocidal properties on the nanocomposites since Cu is known for its antibacterial performance. The effect of the Cu concentration was investigated. The nanocomposites were prepared with high-shear stirring. Specimens were 3D printed following international standards for mechanical testing. Their thermal and spectroscopic response was also investigated. The morphological characteristics were examined. The antibacterial performance was evaluated with an agar well diffusion screening process. The experimental results were analyzed with statistical modeling tools with two control parameters (three levels each) and eleven response parameters. Cu enhanced the mechanical properties in all cases studied. 0.5 wt.% Cu nanocomposite showed the highest improvement (approximately 11% in tensile and 10% in flexural strength). The antibacterial performance was sufficient against *S. aureus* and marginal against *E. coli*.

## 1. Introduction

Vat photopolymerization (VPP) can build accurate and high-resolution parts [1]. It uses photopolymer resins that are converted into a solid part with an Ultra Violet (UV) laser [2]. The mechanical properties of the VPP resins have been studied under different types of tests [1,3,4,5,6]. The process is popular in different industrial fields, such as the automotive industry and the medical sector [7]. Nevertheless, in some cases, the inferior mechanical properties of the 3D-printed parts, compared to the corresponding injection molding parts, restrict the use of the process [8]. This inferior mechanical performance is due to the 3D printing structure. As expected, the effect of the 3D printing parameters on the mechanical properties of the parts built with the process is studied in the literature for different materials (pure and composites), 3D printing parameters, and processes, and the results are correlated and evaluated with the corresponding injection molded parts [9,10,11,12]. The performance of the 3D printed parts is experimentally verified in the literature [13,14,15,16,17].

Research has focused on the materials employed in VPP in an effort to improve their overall performance [7,18,19,20,21]. Quality characteristics of the built parts, such as the surface roughness and glossiness have also been investigated [22]. Parts built with 3D printing processes have such quality issues, therefore their quality characteristics are often studied in the literature for different 3D printing processes as well [23,24]. In VPP AM, another issue of the resins used in VPP is the shrinking of the building part when it is cured, which reduces the overall dimensions of the parts, with research aiming to reduce this effect conducted in the literature, thus improving the quality of the build parts [4,25,26,27]. To improve the performance of the resins, additives have been used and composites have been developed [2,8].

As mentioned, VPP has often been applied in the medical field [5,20,26], with numerous different studies presented in the literature. Cytotoxicity is a common problem [26], which has also been studied for the resins used in dentistry applications [28], in which the VPP process is also popular [25]. Biobased resins have been developed in an effort to better meet the needs of the medical field [21,29,30,31]. VPP has been applied in tissue engineering [30,32,33,34,35], bone scaffolds [36,37,38,39,40], drug delivery, and medical device applications [41,42]. The performance of sterilized resins for surgical implant applications has also been investigated [43]. To enhance the mechanical performance and to induce biocidal properties to the VPP resins, additives have been used, such as Copper (Cu) nanoparticles, and corresponding nanocomposites have been developed [44].

Commercially there are medical-grade resins available that have been employed in different types of medical applications in the literature. Their biocompatibility, how to certify it, and the processing requirements have been presented in the literature [45]. Medical grade resins have been employed in cadavers for pelvic tumor resection [46], for bone fixation in femoral fractures in orthopedics [47], in surgical instruments [48], in face anatomy for the fabrication of face masks [49], in health monitoring devices [50], in tracheal grafts [51], in implantable vaccines [52], in tissues [53], in bio-microfluidic devices [54], in oncological orthopedics [55], in medical inhalers [56], and in eye treatments [57]. Nanocomposites have been developed to enhance the mechanical performance of medical-grade resins [58] and to induce antibacterial properties [59].

Organic materials are used in different types of applications, such as energy [60], storage [61,62], photovoltaic [63], and optoelectronic [64] applications. Their degree of conversion has been often studied for dental applications [65,66,67,68,69,70]. Copper (Cu) is a popular metal, widely used [71], due to its properties, such as high thermal and electrical conductivity, easy processability [72], good corrosion resistance [73], and antibacterial properties against various pathogenic bacteria, such as Gram-positive *Staphylococcus aureus (S. aureus)* and Gram-negative *Escherichia coli (E. coli)* [74]. Therefore, it is used in different industries from automotive to aerospace [72] and medicine [74]. It has been applied for applications, such as catalysis and films [75], microelectronics and electronics, heating [73], electrical applications [71,72], marine applications [76], and medical devices, water and food applications [77]. Research in the medical field in the literature for the reduction of bacteria populations is wide, from marine organisms [78] to bees [79]. In nanoparticle form, Cu has been applied to induce properties in composites, mainly due to its biocidal behavior [71,77,80,81,82,83], with research on the antibacterial performance of the Cu nanoparticles and their use in medical applications being wide [84,85,86]. In AM it has been used in powder-based 3D printing processes to build parts [72,73,87,88], in electron beam melting AM [89], and in ultrasonic AM [90]. In nanoparticle form, Cu has been used to induce antibacterial properties in polymers used as matrix materials in vat photopolymerization [44,91] and in material extrusion (MEX) 3D printing [92,93]. The introduction of antibacterial properties in polymeric materials in different AM processes has gained interest in the research, due to the potential of such composites in medical and food applications. Metal oxide nanoparticles are commonly used to achieve that behavior [80], and apart from Cu_2_O [94] and Cu [95], nanoparticles, such as titanium oxide (TiO_2_) [96], silicon dioxide (SiO_2_) [97], silver [98,99,100,101,102], and carbon-based nanoparticles [103,104], have been investigated, and promising results were reported.

This work presents for the first time in the literature, for this specific medical grade resin in vat photopolymerization AM, the use of Cu nanoparticles for the production of nanocomposites with enhanced mechanical properties and antibacterial properties. Such materials can be used in demanding medical and culinary applications, as it was derived from the literature review presented above. As presented above, organic materials are popular in various fields of medicine and more specifically in the dental sector [65,66,67,68,69,70]. In this work, nanocomposites were developed at different Cu concentrations and tested for their mechanical properties. Their thermal and spectroscopic response was also investigated. Their morphological characteristics were inspected with an optical stereoscope and a field emission Scanning Electron Microscope (SEM). The experimental results were analyzed and optimized with statistical modeling tools. Two control parameters with three levels each were examined against eleven response parameters, related to the quality and the mechanical strength of the samples fabricated with the prepared nanocomposites. Finally, the antibacterial performance was tested against two bacteria (*S. aureus*, and *E. coli*) with an agar well diffusion method. These bacteria were selected as they are the most commonly studied for such types of research [74,81,82,83,105]. It was found that the 0.5 wt.% Cu nanocomposite exhibited the highest mechanical properties among the materials tested, with an approximately 11% increase in the tensile and 10% increase in the flexural strength, compared to samples built with the stock biomed amber resin. The 1.0 wt.% Cu nanocomposite showed better quality characteristics (surface roughness and dimensional accuracy). All nanocomposites exhibited biocidal performance against the *S. aureus* bacterium, while the corresponding biocidal performance against *E. coli* was marginal.

## 2. Materials and Methods

**Figure 1:**
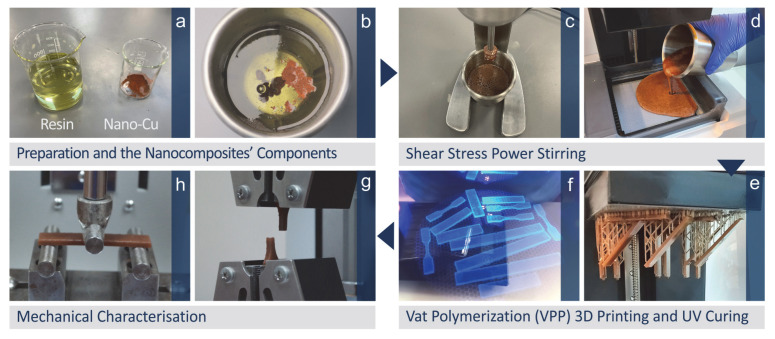
The steps of the methodology followed in the work are (**a**) raw materials, (**b**) mixing of the raw materials in the weight-to-weight concentration, (**c**) shear stress power stirring, (**d**) placing the mixture in the vat photopolymerization 3D printer, (**e**) 3D printing of the specimens for mechanical testing, (**f**) UV curing of the specimens, (**g**) tensile testing, (**h**) three-point-bending flexural test.

Figure 1 presents pictures of the nanocomposites’ preparation and characterization from the raw materials mixture to the fabrication of the samples and their mechanical testing. The methodology of the work is analytically presented further below.

### 2.1. Materials

The vat photopolymerization medical grade resin was procured from Formlabs (Formlabs Ohio Inc., Millbury, OH, USA). A commercial medical grade resin is a biocompatible and biostandable resin, with known specifications and composition, certified by its manufacturer. This medical-grade resin was used as the matrix material in the work. Formlabs (Formlabs Ohio Inc., Millbury, OH, USA) produces different grades of medical resins for VPP. In this work, biomed amber was used, which is a medical grade resin suitable for strong and stiff parts production (ultimate tensile strength—UTS 73 MPa, flexural strength 103 MPa) with expected short-term skin contact. Copper nanoparticles were procured from Nanographi (Nanografi Inc., Ankara, Turkey). Their specifications are size 80–240 nm, purity 99.95%, true density 8.9 g/cm^3^, shape spherical, and specific surface area 4.6 m^2^/g. All data are according to the manufacturers’ datasheets.

### 2.2. Copper Nanoparticles Inspection

Initially, the Cu nanoparticles were examined with Scanning Electron Microscopy (SEM). A Jeol JSM-IT700HR (Jeol Ltd., Tokyo, Japan) field emission SEM was used. Images were taken on non-sputtered powder, aiming to verify the shape and the size of the nanoparticles. Energy Dispersive Spectroscopy (EDS) analysis was also conducted to verify the elements in the powder.

### 2.3. Samples Preparation

Herein, samples for the mechanical, thermal, spectroscopic, and antibacterial tests were prepared from three different nano-compounds (Table 1).

Stock biomed amber parts were 3D printed for comparison purposes, with the same 3D printing settings as the nanocomposites. The same package of samples was prepared with the nanocomposites. Nanocomposites with biomed amber as the matrix material and Cu nanoparticles as the filler were prepared at two different concentrations, 0.5 weight-to-weight (wt.%) and 1.0 wt.%, as mentioned. These concentrations were selected, aiming to determine the effect of Cu at low concentrations. For the preparation of the nanocomposites, the raw materials were weighted at the correct proportions and two different mixtures, one for each nanocomposite, were prepared. Each compound was stirred in a high rotational/shear stirrer to ensure the homogeneous dispersion of the Cu nanoparticles in the biomedical resin, i.e., to produce a grade consistent suspension for both the 0.5 wt.% and 1.0 wt.% filler concentrations. The process followed for the preparation of the nanocomposites is compatible with the literature for the preparation of composites for the VPP process [106,107,108].

Then, each suspension was put in an air vacuum devise in order to be degassed. Each resin/nanocomposite afterward filled properly in the tank of the VPP 3D printer device to produce the corresponding 3D printing samples. A Formlabs Form 3B (Formlabs Ohio Inc., Millbury, OH, USA) VPP 3D printer was used for the fabrication of the samples. Tensile (type V specimens with 3.2 mm height, according to the ASTM D638-14 international standard) and flexural specimens (according to the ASTM D790-10 international standard) were 3D printed, along with cylindrical specimens (ø12 mm × 4 mm) for the antibacterial tests. Six specimens were fabricated in each case. Specimens were built at 45 degree angles to the build platform. The 3D printing settings were selected according to the default settings of the 3D printer for this specific resin to achieve good quality parts (layer thickness 0.05 mm). After the completion of the build process, parts were washed in a Formlabs Form Wash machine (Formlabs Ohio Inc., Millbury, OH, USA) for 20 min. A Formlabs Form Cure (Formlabs Ohio Inc., OH, USA) machine was used for the curing of the parts, according to the manufacturer’s instructions and the literature on the specific resin [27].

### 2.4. Thermal and Spectroscopic Analysis

Thermogravimetric analysis (TGA) measurements (Perkin Elmer Diamond, Perkin Elmer Inc., Waltham, MA, USA, 30–550 °C, step 10 °C/min, a Nitrogen atmosphere) were taken on samples of approximately 10 mg, taken from the 3D printed samples, to determine the thermal properties and stability of the produced nanocomposites.

Raman measurements were performed with a modified LabRAM HR Raman Spectrometer (HORIBA Scientific, Kyoto, Japan). Raman excitation was achieved with a 532 nm central wavelength solid-state laser module with a maximum laser output power of 90 mW. The microscope is coupled with a 50× microscopic objective lens with a 0.5 numerical aperture and 10.6 mm working distance (LMPlanFL N, Olympus) that delivered the excitation light and collected the Raman signals. A neutral density filter of 5% transmittance was used, which resulted in 2 mW of power on the sample. The laser spot size was approximately 1.7 μm laterally and approximately 2 μm axially. A 600 grove grating was used, resulting in a Raman spectral resolution of approximately 2 cm^−1^. The Raman spectral range was set to be from 500 to 3900 cm^−1^, resulting in 3 optical windows per point. The acquisition time for each measurement was 10 s and with five accumulations at each point.

### 2.5. Quality Characteristics and Mechanical Characterization

For the evaluation of the quality of the 3D printed parts, surface roughness measurements were taken in the longitudinal and the vertical direction with a Taylor Hobson surtronic3+ (Taylor Hobson, Leicester, UK) roughness gage device. A common issue in parts made with VPP is the shrinkage of the parts [4,25,26,27], therefore the dimensions of the section of the tensile parts in the middle of the part were measured with a high-quality caliper, and the effect of the control parameters (Cu concentration in the nanocomposites and 3D printing direction) studied in the work was evaluated.

For the evaluation of the control parameters in the mechanical strength of the 3D printed parts, tensile and flexural tests were conducted. Experiments were carried out on an Imada MX2 (Imada Inc., Northbrook, IL, USA) machine, according to the corresponding standards (ASTM D638-14 for the tensile tests and ASTM D790-10 for the flexural tests). In each type of experiment, the machine had a suitable setup for the test. In the tensile tests, standardized grips were used, while in the flexural tests, a three-point-bending setup was used with a 52 mm span, following the corresponding standard. Elongation speeds were set at 10 mm/min and experiments were carried out at room temperature. Five specimens were tested in each type of experiment, out of the six manufactured in the VPP 3D printing process.

### 2.6. Morphological Examination

The 3D printed specimens’ morphological characteristics were examined with an optical stereoscope (KERN OZR5, equipped with a KERN ODC 832 5MP camera, KERN & SOHN GmbH, Balingen, Germany) and a Jeol JSM-IT700HR (Jeol Ltd., Tokyo, Japan) field emission SEM. Images were taken on the side of the tensile specimens, to evaluate the 3D printing quality and to locate possible defects. The fracture surface of tensile specimens was also inspected with the same SEM at different magnifications to evaluate the fracture mechanism and to locate possible agglomerations of Cu in the nanocomposites.

### 2.7. Design of Experiment, Statistical Analysis, and Optimization of the Experimental Results

For the analysis and optimization of the experimental findings a 3 × 3 full factorial array was formed. The control parameters were the Cu concentration in the materials and the 3D printing angle of the samples, with three levels each (Table 2). The response parameters were related to the quality characteristics of the samples, i.e., cross-sectional area (mm^2^) and its (%) deviation to the nominal value; average surface roughness Ra (μm) in the horizontal and in the vertical direction; and surface roughness Rz (μm) in the horizontal and in the vertical direction, as well as to the mechanical properties of the samples, i.e., tensile strength (MPa), the tensile modulus of elasticity (GPa); tensile toughness (MJ/m^3^); flexural strength (MPa); the flexural modulus of elasticity (GPa); and flexural toughness (MJ/m^3^). Results were statistically analyzed, an Analysis of Variances (ANOVA) followed, and modeling equations were produced. The effect of the control parameters in the response parameters is presented and analyzed further down in the work in the results section.

### 2.8. Antibacterial Performance of the Nanocomposites

A screening agar well diffusion method was used to evaluate the ability of the Cu nanoparticles to induce antibacterial properties in the biomed amber resin, with the process followed for the development of the nanocomposites. The negative control in the work was the sample made with pure biomed resin. The positive controls for the biological experiment were the samples made with the two nanocomposites in the work (nanocomposite with 0.5 wt.% and 1.0 wt.% Cu nanoparticles concentration).

The McFarland Standard 0.5 was followed in the work. The biocidal performance of the nanocomposites was tested against Gram-positive *Staphylococcus aureus (S. aureus)* and Gram-negative *Escherichia coli (E. coli)* bacteria. Bacteria we certified and sourced by the local microbiologist’s association (Hellenic Cooperative of Laboratory Doctors—MEDISYN, Athens, Greece, https://www.medisyn.eu/en/homepage, accessed on 1 November 2022). The 3D printed nanocomposites (with cylindrical shape and dimensions Ø12 mm × 4 mm) were placed in two types of 85 mm Petri dishes, each having suitable growth material for each bacterium. Bacterium growth was performed at 37 °C for 24 h. The ability of the nanocomposites prepared in the work to inhibit growth of the bacteria was evaluated by the inhibition zones (IZ) developed on the Petri dishes after the 24 h period. The IZ was measured with an optical microscope (Kern OKO 1, equipped with a KERN ODC 832 5MP camera, KERN & SOHN GmbH, Balingen, Germany).

## 3. Results

### 3.1. Copper Nanoparticles Inspection

**Figure 2:**
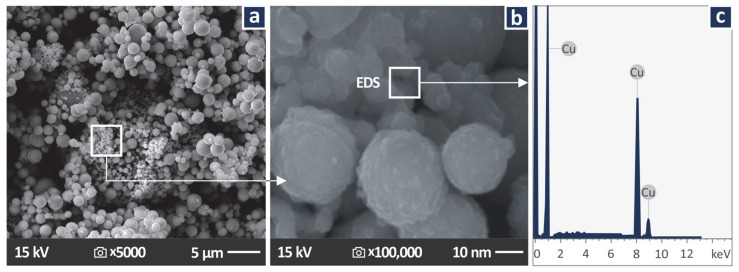
Cu nanopowder: (**a**) 5000× SEM image of the Cu nanoparticles, (**b**) 100,000× SEM image of the Cu nanoparticles, (**c**) EDS graph on the region shown in (**b**).

Figure 2 presents SEM images taken on the powder used as the additive in the work (Cu nanoparticles). In the images taken, the shape and the size of the nanoparticles were verified, with only very few nanoparticles observed with dimensions higher than the manufacturer’s specs. Figure 2c presents the Energy Dispersive Spectroscopy (EDS) graph produced by examining the region shown in Figure 2b. As expected, high peaks of the Cu element are shown, indicating a high concentration of the element in the observation region.

### 3.2. Thermal and Spectroscopic Analysis

**Figure 3:**
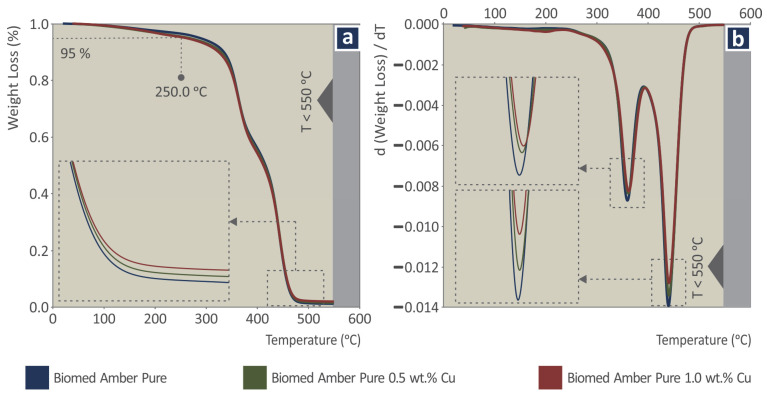
(**a**) Weight loss vs. temperature graph, (**b**) Weight loss rate vs. temperature graph.

Figure 3a presents the weight loss vs. temperature graph for each one of the materials studied in the work (stock biomed amber and biomed amber/Cu nanocomposites at two Cu concentrations), as they were produced in the thermogravimetric analysis (TGA) measurements, that is, the degradation of the material (loss of the mass) with the increase in the temperature, as it was derived from the TGA measurements. From the graphs, it is shown that the materials have a similar thermal response. The degradation of the materials starts at approximately 250 °C. The thermal stability of the biomed amber resin was not affected by the addition of the Cu nanoparticles. The remaining material quantities after the completion of the measurements agree with the concentration of the Cu additive in the corresponding nanocomposites. Figure 3b presents the corresponding weight loss rate vs. temperature graphs, i.e., how fast the mass degradation occurred in the experiment and how this rate changes with the increase of the temperature. The maximum weight loss rate occurs at roughly the same temperature for all materials. The weight loss rate decreases with the increase of the Cu loading in the nanocomposites, with the lowest value depicted for the 1 wt. % Cu nanocomposite. This is the expected response, as with the increase of the Cu loading, the mass that does not degrade during the TGA measurement increases. The remaining mass after the completion of the TGA measurements agrees with the Cu concentration in each nanocomposite.

**Figure 4:**
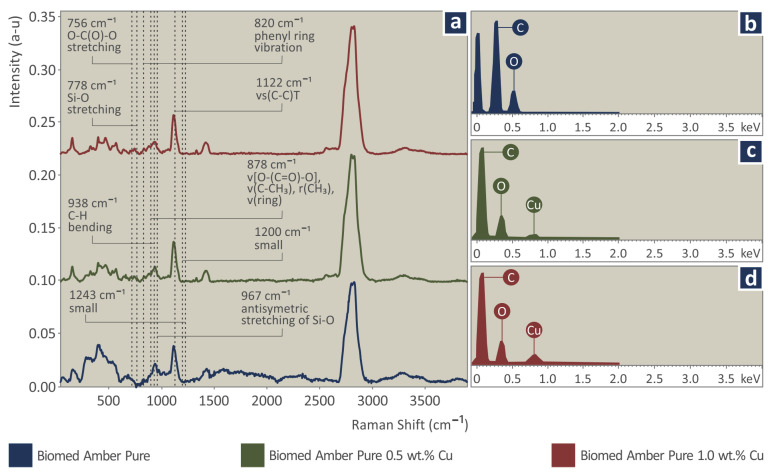
(**a**) Raman spectra, EDS graphs (**b**) Biomed amber pure, (**c**) Biomed amber 0.5 wt.% Cu, (**d**) Biomed amber 1.0 wt.% Cu. The legend shows which graph corresponds to which material, according to the color of the graph.

As is seen in Figure 4, the major Raman peaks are from amber pure. The addition of Cu in amber samples presented a gradual drop in the photoluminescence together with an increase in the density of Cu particles. This caused a clearer Raman signal in the range between 500 and 1300 cm^−1^. From the analysis of amber pure, the major Raman peaks are identified, and their related assignments are presented in the following Table 3. The range of the Raman peaks found is between 500 cm^−1^ and 3000 cm^−1^. The Si-O peaks are from the resin since they appear in all the observations in the work. Throughout the analysis, the main compositional influence is the addition of the Cu, which at such small concentrations does not affect the observed peaks in the Raman spectra, with changes in the small peaks being marginal. It should be noted that there was no visible or measurable effect from the laser irradiation on the sample, during Raman signal acquisition.

The following Raman peaks increased with the addition of Cu particles, most probably due to the photoluminescence signal drop, and are shown in the next Table 4.

### 3.3. Mechanical Characterization

**Figure 5:**
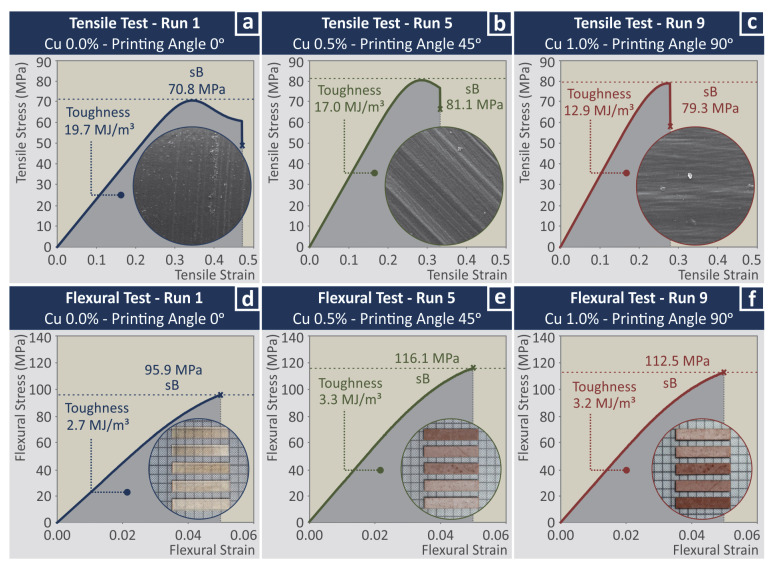
Tensile and flexural stress vs. strain graphs from one randomly selected specimen for each case (**a**) tensile test of biomed amber pure 3D printed at 0 degree angle, (**b**) tensile test of biomed amber 0.5 wt.% Cu, 3D printed at 45 degree angle, (**c**) tensile test of biomed amber 1.0 wt.% Cu, 3D printed at 90 degree angle, (**d**) flexural test of biomed amber pure 3D printed at 0 degree angle, (**e**) flexural test of biomed amber 0.5 wt.% Cu, 3D printed at 45 degree angle, (**f**) flexural test of biomed amber 1.0 wt.% Cu, 3D printed at 90 degree angle.

As mentioned, for the mechanical characterization of the nanocomposites, tensile and three-point bending flexural tests were carried out. Five specimens were tested for each different case studied (levels of wt.% concentration and printing angle—PA). Figure 5 presents a typical graph of a randomly selected specimen from each different sample studied (wt.% concentration and printing angle—PA). Tensile and flexural stress vs. strain graphs is presented, which were produced during the experiments. The purpose of Figure 5 is to show one of the five graphs produced in each test for three samples prepared with different parameters (first run, middle run, and last run). The aim is to show the effect of the different control parameter values on the mechanical behavior of the nanocomposites and how the different nanocomposites behave in the mechanical tests. In the inset images in the figures, microscope images from the side of the samples indicating the PA of the specimen presented in the corresponding graph are shown. In the curves produced in the flexural tests, randomly selected specimens are shown. The corresponding toughness values calculated as the integral of the stress vs. strain graph are also mentioned in each graph. The average mechanical properties calculated for each case from the experiments are presented in the statistical analysis section of the work in comparison with the corresponding control parameters.

### 3.4. Morphological Examination

**Figure 6:**
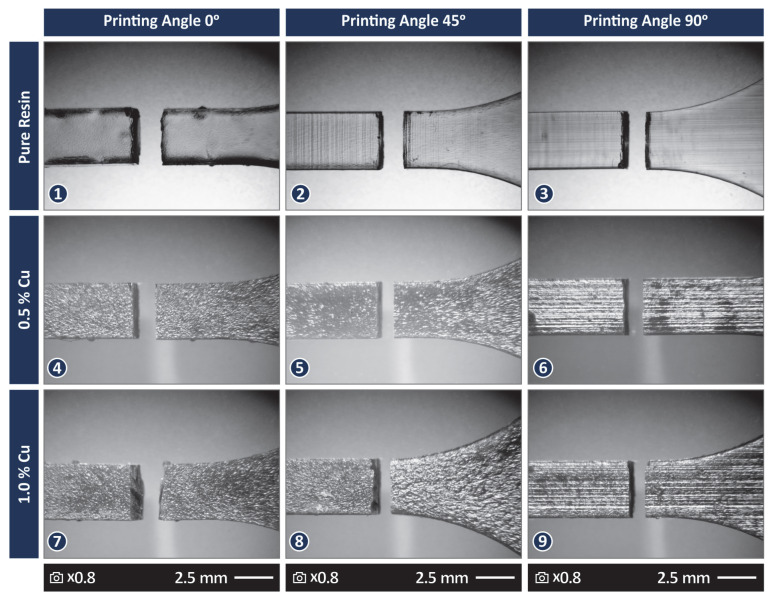
Stereoscope images at 0.8× magnification (**1**) Biomed amber pure, 0 degree 3D printing angle, (**2**) Biomed amber pure, 45 degree 3D printing angle, (**3**) Biomed amber pure, 90 degree 3D printing angle, (**4**) Biomed amber 0.5 wt.% Cu, 0 degree 3D printing angle, (**5**) Biomed amber 0.5 wt.% Cu, 45 degree 3D printing angle, (**6**) Biomed amber 0.5 wt.% Cu, 90 degree 3D printing angle, (**7**) Biomed amber 1.0 wt.% Cu, 0 degree 3D printing angle, (**8**) Biomed amber 1.0 wt.% Cu, 45 degree 3D printing angle, (**9)** Biomed amber 1.0 wt.% Cu, 90 degree 3D printing angle.

Figure 6 presents stereoscopic images from the top of the tensile samples after they fail the tests. One random sample from each different case study is presented. From the images, a qualitative evaluation can be made. The samples manufactured with the stock biomed amber material show slightly higher deformation in their fracture area when compared with the samples made with the nanocomposites, still a brittle failure is observed. This agrees with the stress vs. strain graphs presented in Figure 5. Regarding the quality of the surface, visually in the images, the samples made with the stock biomed amber material are less rough than the samples made with the nanocomposites. The samples made with the nanocomposite with 0.5 wt.% Cu show the roughest surfaces among the samples tested.

**Figure 7:**
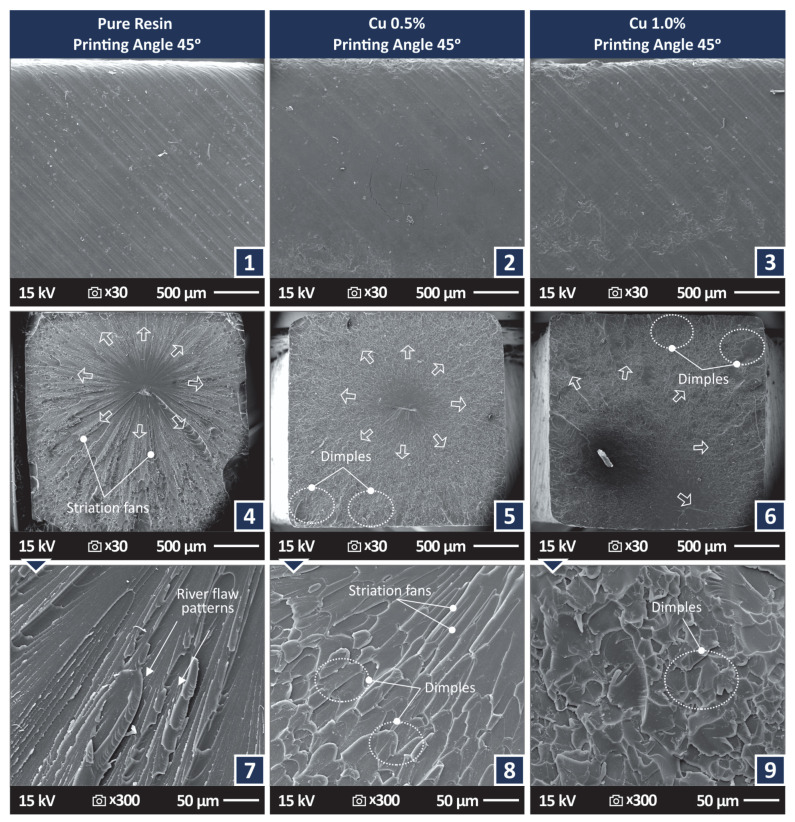
SEM images for samples 3D printed with 45 degree angles. Side surface at 30× magnification (**1**) Biomed amber pure, (**2**) Biomed amber 0.5 wt.% Cu, (**3**) Biomed amber 1.0 wt.% Cu, Fracture surface at 30× magnification (**4**) Biomed amber pure, (**5**) Biomed amber 0.5 wt.% Cu, (**6**) Biomed amber 1.0 wt.% Cu, Fracture surface at 300× magnification (**7**) Biomed amber pure, (**8**) Biomed amber 0.5 wt.% Cu, (**9**) Biomed amber 1.0 wt.% Cu. The arrows show the beginning of the fracture and how it evolved.

Figure 7(1)–(3) present side surface SEM images from tensile samples 3D printed with 45 degree angles with the different materials tested. The PA can be observed in the SEM images. In all samples, a 3D printing structure with no defects or voids can be observed. The remaining images of Figure 7 present the corresponding fracture area images for the aforementioned samples of Figure 7 at two different magnifications. In all samples, the evolution of the fracture during their failure is observed and indicated in the figures. The characteristic striation lines, river flow pattern, and dimples of the polymers in vat photopolymerization are observed in the figure [116].

For the sample made with the stock biomed amber material and the sample made with the 0.5 wt.% nanocomposite, the fracture started at approximately the center of the samples’ cross-section area. For the sample made with the 1.0 wt.% nanocomposite, the fracture start was off-centered. In the higher magnification images, the fracture area of the sample made with the stock biomed amber material (Figure 7(7)) shows some deformation, while the samples made with the two nanocomposites show a more brittle fracture mechanism, which agrees with the experimental graphs and the observations in the optical stereoscope.

**Figure 8:**
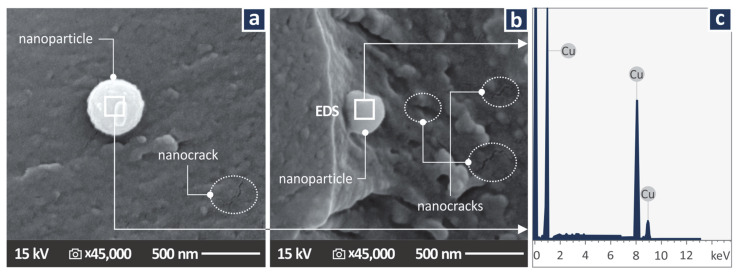
SEM images at 45,000× magnification on the fracture surface of tensile test samples made with the nanocomposites prepared in the work (**a**) Biomed amber 0.5 wt.% Cu, (**b**) Biomed amber 1.0 wt.% Cu, (**c**) EDS graph acquired on the region indicated in (**b**).

Figure 8 shows higher magnification SEM images of 45,000× for the two nanocomposites studied. Images were also taken on the fracture surface of the tensile test samples. In these nanoscale images, no agglomerations were located in the samples, while nanoparticles were identified. Additionally, nano-cracks on the samples were spotted in the fracture area, showing somehow that the failure of the specimen was not restricted only to the fracture area, but also slightly diffused in the remaining sample’s structure. Energy Dispersive Spectroscopy (EDS) graphs were produced by analyzing regions of the fracture area in which nanoparticles were observed (Figure 8c). The EDS graphs verified the existence of nanoparticles, as the peaks of the Cu element in the produced graphs were high, indicating a high concentration of the element in the observed regions. No unexpected elements were identified in the EDS graphs and no nanoparticles agglomerations were located.

### 3.5. Statistical Analysis and Optimization of the Experimental Results

Table 5 and Table 6 present the average values and the deviation for the response parameters of the study related to the quality characteristics and the mechanical properties, respectively, for each experimental run. The analytic experimental results are presented in Tables S1 and S2 of the supplementary material.

**Figure 9:**
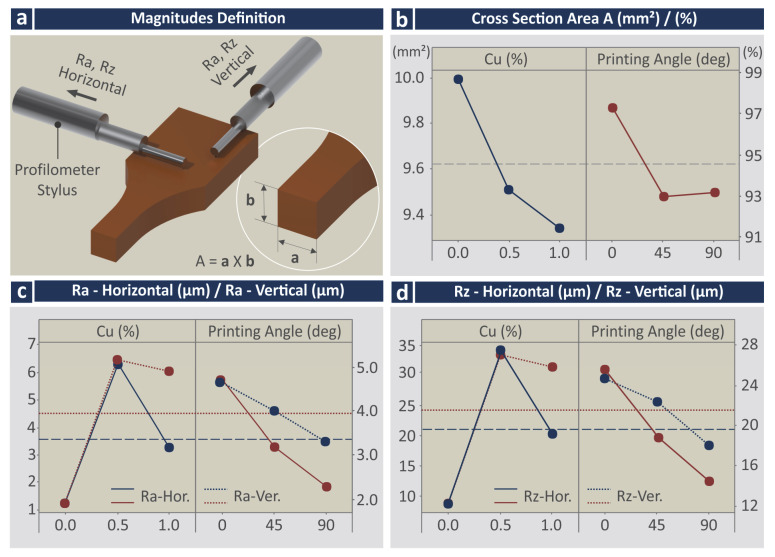
Main effects plots vs. control parameters (Cu concentration and printing angle) (**a**) surface roughness and area measurements’ location, (**b**) cross-section area (mm^2^/%), (**c**) surface roughness Ra (μm) horizontal and vertical measurements, (**d**) surface roughness Rz (μm) horizontal and vertical measurements.

Figure 9 presents the Main Effect Plots (MEPs) for the response parameters related to the quality characteristics of the 3D printed specimens. Figure 9a illustrates how the surface roughness measurements were taken and what was measured for the cross-section area calculation in the specimens (measurements were taken with a high-quality caliper). It should be mentioned that VPP 3D parts exhibit shrinkage issues, so it was not expected to measure the dimensions of the manufactured parts higher than the nominal. Shrinkage occurred in both directions measured in the section area (not in the same amount).

In the MEPs for the cross-section area and the (%) deviation from the nominal value (Figure 9b), it can be observed that the shrinkage of the specimens is increased with the increase of the Cu concentration on the materials. Regarding the printing angle (PA), the specimens built with 0 degrees PA depict the lowest shrinkage, while the specimens built with a PA of 45- and 90-degrees depict increased shrinkage values. Regarding the surface roughness Ra, samples built with the stock biomed amber material exhibited the lowest Ra values in both measured directions, while samples built with the 0.5 wt.% Cu concentration nanocomposite exhibited the highest Ra values in both measured directions. Samples built with the 1.0 wt.% Cu nanocomposite show decreased values, but still higher than the samples built with the 0.5 wt.% Cu concentration nanocomposite. This agrees with the findings from the visual inspection of the samples in the optical stereoscope (Figure 6). Surface roughness measurements follow the same trend regarding their values in both measured directions. Regarding the surface roughness Ra vs. the PA, the 0 degrees sample showed the highest Ra values, with Ra decreasing with the increase of the PA. The same trend and observations can be made regarding the values of the measured surface roughness Rz (Figure 9d).

**Figure 10:**
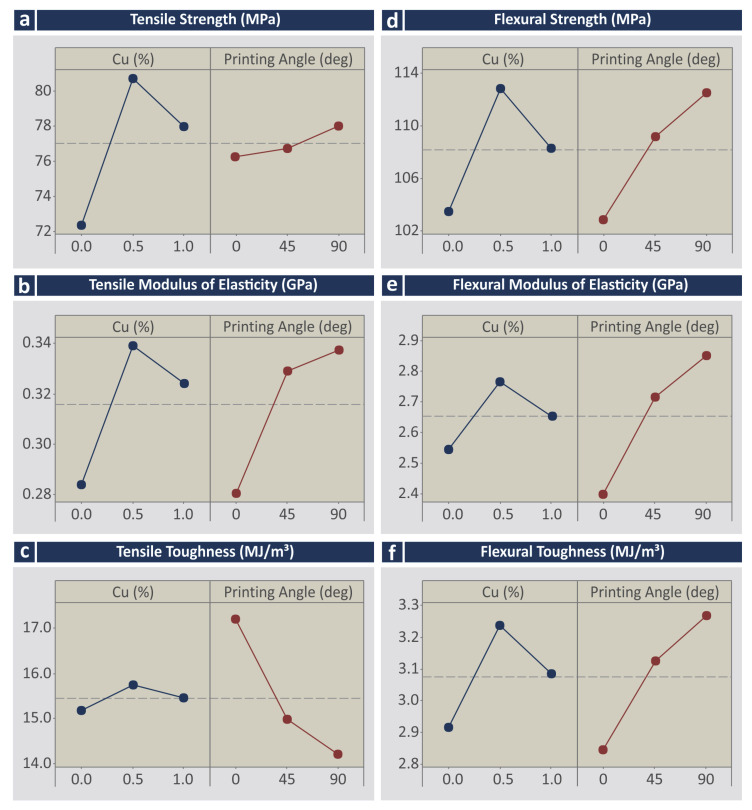
Main effects plots vs. control parameters (Cu concentration and printing angle) (**a**) tensile strength (MPa), (**b**) tensile modulus of elasticity (GPa), (**c**) tensile toughness (MJ/m^3^), (**d**) flexural strength (MPa), (**e**) flexural modulus of elasticity (GPa), (**f**) flexural toughness (MJ/m^3^).

The corresponding MEPs for the response parameters related to the mechanical properties of the 3D-printed specimens are presented in Figure 10. As it can be observed, all of the response parameters (tensile strength—MPa, the tensile modulus of elasticity (GPa), tensile toughness (MJ/m^3^), flexural strength (MPa), the flexural modulus of elasticity (GPa), and flexural toughness (MJ/m^3^) follow the same trend regarding the Cu concentration on the materials. The addition of Cu increased the mechanical properties, with the highest improvement observed on the samples built with the 0.5 wt.% Cu concentration nanocomposite. The samples built with the 1.0 wt.% Cu concentration nanocomposite exhibited reduced mechanical properties that were still higher than the samples built with the stock biomed amber material. PA has no significant effect on tensile strength (Figure 10a). The increase of PA increases the tensile modulus of elasticity (GPa) (Figure 10b), the flexural modulus of elasticity (GPa) (Figure 10e), and the flexural toughness (MJ/m^3^) (Figure 10f), while it reduces the tensile toughness (MJ/m^3^) (Figure 10c).

**Figure 11:**
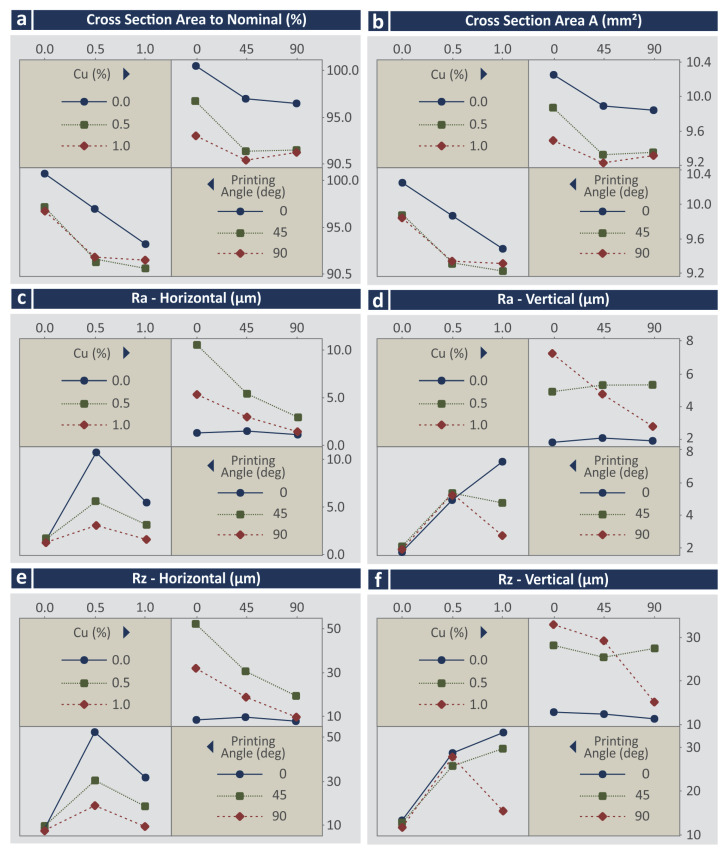
Interaction plots vs. control parameters (Cu concentration and printing angle) (**a**) cross-section area to nominal (%), (**b**) cross-section area (mm^2^), (**c**) surface roughness Ra (μm) horizontal, (**d**) surface roughness Ra (μm) vertical, (**e**) surface roughness Rz (μm) horizontal, (**f**) surface roughness Rz (μm) vertical.

**Figure 12:**
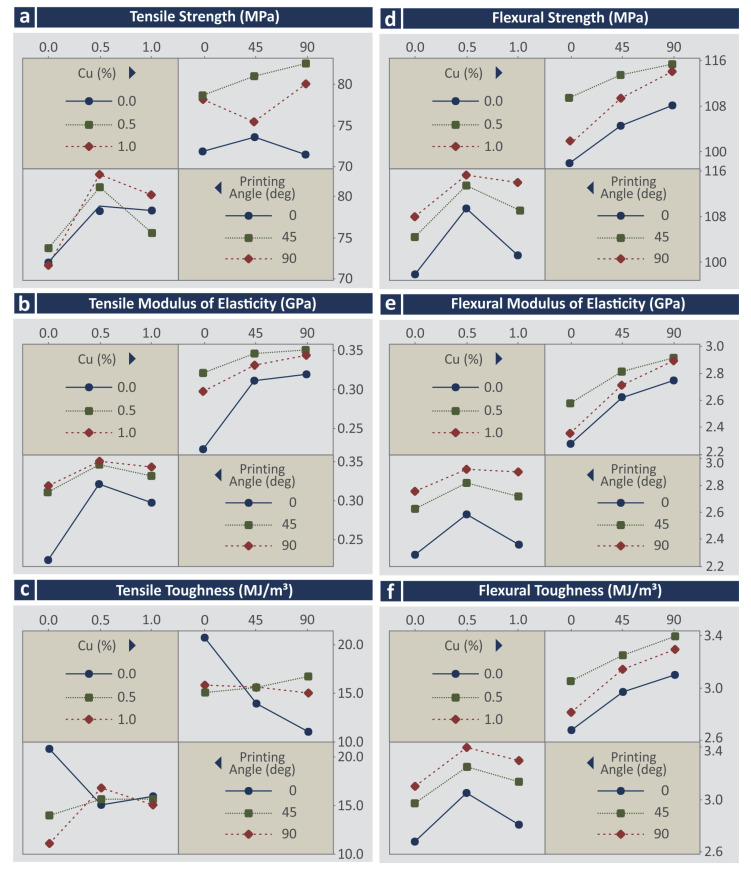
Interaction plots vs. control parameters (Cu concentration and printing angle) (**a**) tensile strength (MPa), (**b**) tensile modulus of elasticity (GPa), (**c**) tensile toughness (MJ/m^3^), (**d**) flexural strength (MPa), (**e**) flexural modulus of elasticity (GPa), (**f**) flexural toughness (MJ/m^3^).

To evaluate the synergistic and antagonistic mechanisms of the control parameters, the corresponding interaction plots were compiled (Figure 11 and Figure 12). Regarding the response parameters related to the quality characteristics of the specimens (Figure 11), in most cases, synergistic interactions can be observed between the control parameters. Only in the cases of Ra and Rz measured in the vertical direction were antagonistic relations found. The interaction plots for the mechanical properties vs. the control parameters (Figure 12) revealed antagonistic interactions for most of the tensile properties, except the tensile modulus of elasticity vs. Cu (%) in which synergistic relations were observed. For the flexural properties, synergistic relations among the control parameters were observed.

### 3.6. Regression and ANOVA

The Reduced Quadratic Regression Model (RQRM) for each response is calculated, according to the following Equation (1):

(1) $$Y_{k}=a_{k}+\overset{n}{\underset{i=1}{\sum }}b_{i,k}x_{i}+\overset{n}{\underset{i=1}{\sum }}c_{i,k}x_{i}^{2}+e_{k}$$

where *k* represents the quality output (e.g., tensile strength, tensile modulus of elasticity, tensile toughness, flexural strength, flexural modulus of elasticity, flexural toughness, tensile specimen measured area, tensile specimen area to nominal area, horizontal and vertical Ra and Rz roughness), a is the constant value, b the coefficient of the linear terms, c the coefficients of the quadratic terms, e the error and *x_i_* the two (*n* = 2) control parameters, i.e., the input 3D printing independent variables.

The ANOVA table for each of the response parameters vs. the control parameters is presented in Tables S3–S14 in the Supplementary Materials of the work. In most cases, the regression values are close to or even higher than 80%, while in the case of the flexural modulus of elasticity (GPa), the flexural toughness (MJ/m^3^), the cross-section area (mm^2^), and the cross-sections area deviation (%), the regression values are higher than 90%, indicating that the models are adequate for the prediction of the corresponding response parameters. Only in the case of the tensile toughness (MJ/m^3^) are the regression values low (~36%), indicating that in this case the model failed to predict the response parameter and cannot be considered reliable for such calculations. For each response parameter, an equation was formed as a function of the control parameters and the equations are presented below:

(2) $$\begin{array}{cc} Area\_Tension= & 10.3053-1.403\cdot \%\mathrm{Cu}\;-0.01445\cdot \mathrm{PA}\;+0.630\\ & \cdot {\%\mathrm{Cu}}^{2}+0.000100\cdot {\mathrm{PA}}^{2}+0.002617\cdot \%\mathrm{Cu}\cdot \mathrm{PA} \end{array}$$

(3) $$\begin{array}{cc} Area\_2\_Nom= & 101.270-13.79\cdot \%\mathrm{Cu}\;-0.1420\cdot \mathrm{PA}\;+6.19\cdot {\%\mathrm{Cu}}^{2}\\ & +0.000986\cdot {\mathrm{PA}}^{2}+0.02572\cdot \%\mathrm{Cu}\cdot \mathrm{PA} \end{array}$$

(4) $$\begin{array}{cc} Ra\_Hor= & 2.465+19.92\cdot \%\mathrm{Cu}\;-0.0436\cdot \mathrm{PA}\;-16.08\cdot {\%\mathrm{Cu}}^{2}\\ & +0.000239\cdot {\mathrm{PA}}^{2}-0.0422\cdot \%\mathrm{Cu}\cdot \mathrm{PA} \end{array}$$

(5) $$\begin{array}{cc} Ra\_Ver= & 1.388+12.36\cdot \%\mathrm{Cu}\;+0.0130\cdot \mathrm{PA}\;-7.05\cdot {\%\mathrm{Cu}}^{2}\\ & -0.000025\cdot {\mathrm{PA}}^{2}-0.05118\cdot \%\mathrm{Cu}\cdot \mathrm{PA} \end{array}$$

(6) $$\begin{array}{cc} Ra\_Ver= & 1.388+12.36\cdot \%\mathrm{Cu}\;+0.0130\cdot \mathrm{PA}\;-7.05\cdot {\%\mathrm{Cu}}^{2}\\ & -0.000025\cdot {\mathrm{PA}}^{2}-0.05118\cdot \%\mathrm{Cu}\cdot \mathrm{PA} \end{array}$$

(7) $$\begin{array}{cc} Rz\_Hor= & 13.22+100.4\cdot \%\mathrm{Cu}\;-0.168\cdot \mathrm{PA}\;-78.1\cdot {\%\mathrm{Cu}}^{2}\\ & +0.00092\cdot {\mathrm{PA}}^{2}-0.2422\cdot \%\mathrm{Cu}\cdot \mathrm{PA} \end{array}$$

(8) $$\begin{array}{cc} Rz\_Ver= & 11.19+53.57\cdot \%\mathrm{Cu}\;+0.0617\cdot \mathrm{PA}\;-31.87\cdot {\%\mathrm{Cu}}^{2}\\ & -0.000502\cdot {\mathrm{PA}}^{2}-0.1816\cdot \%\mathrm{Cu}\cdot \mathrm{PA} \end{array}$$

(9) $$\begin{array}{cc} sB\_Tension= & 72.171+26.71\cdot \%\mathrm{Cu}\;-0.0109\cdot \mathrm{PA}\;-22.27\cdot {\%\mathrm{Cu}}^{2}\\ & +0.000201\cdot {\mathrm{PA}}^{2}+0.0250\cdot \%\mathrm{Cu}\cdot \mathrm{PA} \end{array}$$

(10) $$\begin{array}{cc} E\_Tension= & 235.54+206.4\cdot \%\mathrm{Cu}\;+1.821\cdot \mathrm{PA}\;-140.5\cdot {\%\mathrm{Cu}}^{2}\\ & -0.00998\cdot {\mathrm{PA}}^{2}-0.574\cdot \%\mathrm{Cu}\cdot \mathrm{PA}[M29] \end{array}$$

(11) $$\begin{array}{cc} Toughness\_Tension & \\ & =19.14-2.56\cdot \%\mathrm{Cu}\;-0.1138\cdot \mathrm{PA}\;-1.63\cdot {\%\mathrm{Cu}}^{2}\\ & +0.000348\cdot {\mathrm{PA}}^{2}+0.0988\cdot \%\mathrm{Cu}\cdot \mathrm{PA} \end{array}$$

(12) $$\begin{array}{cc} sB\_Flexural= & 98.813+31.13\cdot \%\mathrm{Cu}\;+0.1568\cdot \mathrm{PA}\;-27.67\cdot {\%\mathrm{Cu}}^{2}\\ & -0.000711\cdot {\mathrm{PA}}^{2}+0.0285\cdot \%\mathrm{Cu}\cdot \mathrm{PA} \end{array}$$

(13) $$\begin{array}{cc} E\_Flexural= & 2309.9+736.1\cdot \%\mathrm{Cu}\;+8.51\cdot \mathrm{PA}\;-666.7\cdot {\%\mathrm{Cu}}^{2}\\ & -0.0433\cdot {\mathrm{PA}}^{2}+0.782\cdot \%\mathrm{Cu}\cdot \mathrm{PA} \end{array}$$

(14) $$\begin{array}{cc} Toughness\_Flexural & \\ & =2.7006+1.0768\cdot \%\mathrm{Cu}\;+0.007251\cdot \mathrm{PA}\;-0.9417\\ & \cdot {\%\mathrm{Cu}}^{2}-0.000032\cdot {\mathrm{PA}}^{2}+0.000711\cdot \%\mathrm{Cu}\cdot \mathrm{PA} \end{array}$$

To identify the statistical importance of each parameter, Pareto charts were formed for all the response parameters (Figures S1–S5 of the Supplementary Materials). The following statistically important parameters were found:

- All parameters for the cross-section area to nominal (%);
- All parameters for the cross-section area (mm^2^);
- Cu, Cu^2^, and Cu × Deg for the Ra in the horizontal direction;
- Cu, Cu^2^, and Cu × Deg for the Ra in the vertical direction;
- Cu, Cu^2^, and Cu × Deg for the Rz in the horizontal direction;
- Cu, Cu^2^, and Cu × Deg for the Rz in the vertical direction;
- Cu and Cu^2^ for the tensile strength (MPa);
- All parameters for the tensile modulus of elasticity (GPa);
- Cu×Deg and Deg for the tensile toughness (MJ/m^3^);
- Cu, Cu^2^, and Deg for the flexural strength (MPa);
- Cu, Cu^2^, Deg, and Deg^2^ for the flexural modulus of elasticity (GPa);
- Cu, Cu^2^, Deg, and Deg^2^ for the flexural toughness (MJ/m^3^).

Additionally, for each response parameter comparative, experimental vs. calculated graphs were formed and the mean absolute percentage error (MAPE) and the Durbin–Watson factor (a measurement indicator of the autocorrelation in the residuals) were calculated to identify the reliability of the equations’ results. In all response parameters, the MAPE calculated values were very acceptable (<2% and in the case of the tensile modulus of elasticity 4.15%), except, as expected the case of the tensile toughness (MJ/m^3^) in which the calculated MAPE value was higher (15.14%), still within acceptable limits. The calculated Durbin–Watson factors ideally should be (>2, <3) and that was the case for most response parameters, or values lower but close to 2 were calculated, which is acceptable for all response parameters.

**Figure 13:**
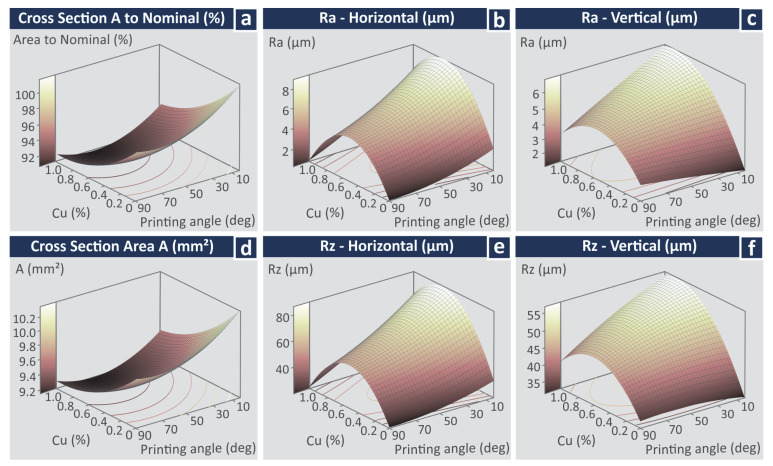
Surface graphs vs. control parameters (Cu concentration and printing angle) (**a**) cross-section area to nominal (%), (**b**) cross-section area (mm^2^), (**c**) surface roughness Ra (μm) horizontal, (**d**) surface roughness Ra (μm) vertical, (**e**) surface roughness Rz (μm) horizontal, (**f**) surface roughness Rz (μm) vertical.

**Figure 14:**
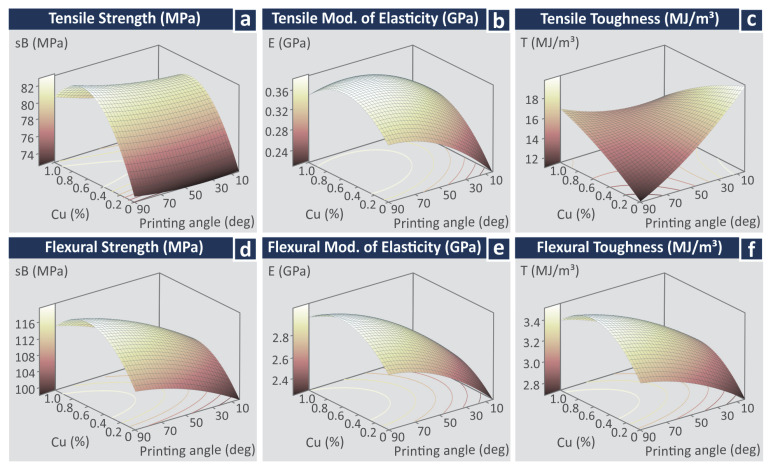
Surface graphs vs. control parameters (Cu concentration and printing angle) (**a**) tensile strength (MPa), (**b**) tensile modulus of elasticity (GPa), (**c**) tensile toughness (MJ/m^3^), (**d**) flexural strength (MPa), (**e**) flexural modulus of elasticity (GPa), (**f**) flexural toughness (MJ/m^3^).

Figure 13 and Figure 14 present the experimental findings of the work as surface graphs for the response vs. the control parameters studied. Figure 13 contains the surface graphs for the response parameters related to the quality characteristics of the samples, while Figure 14 depicts the response parameters related to the mechanical properties vs. the control parameters.

### 3.7. Antibacterial Performance of the Nanocomposites

**Figure 15:**
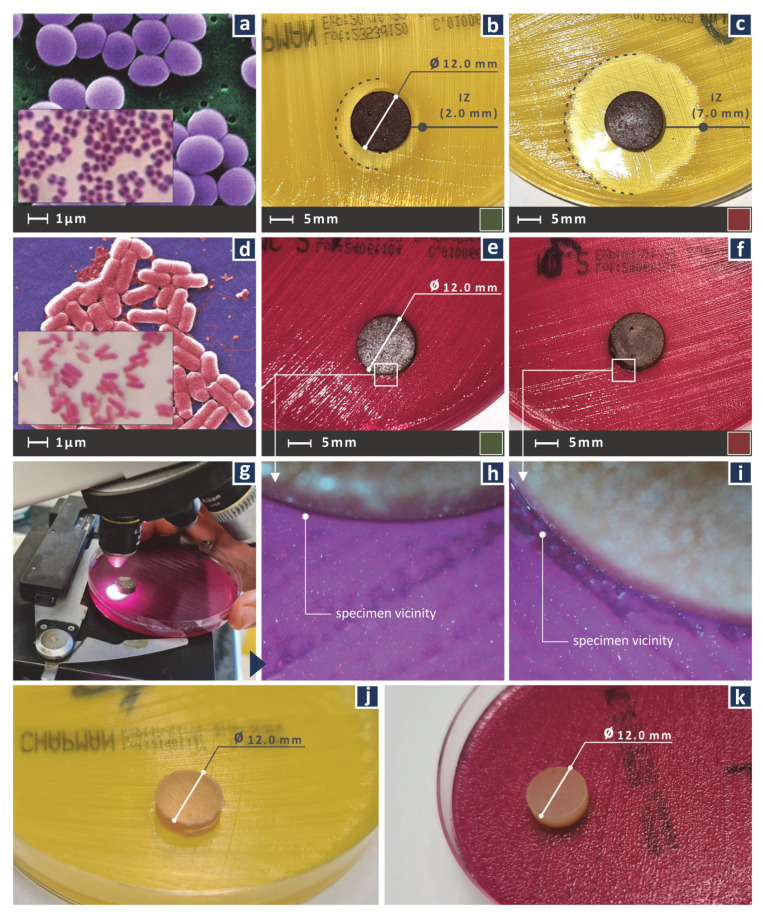
(**a**) *S. aureus*, (**b**) inhibition zone of the biomed amber 0.5 wt.% Cu developed in the *S. aureus* culture, (**c**) inhibition zone of the biomed amber 1.0 wt.% Cu developed in the *S. aureus* culture, (**d**) *E. coli*, (**e**) inhibition zone of the biomed amber 0.5 wt.% Cu developed in the *E. coli* culture, (**f**) inhibition zone of the biomed amber 1.0 wt.% Cu developed in the *E. coli* culture, (**g**) inspection on the microscope of the specimen in the Petri dish with the *E. coli* culture, (**h**) higher magnification microscope image of the biomed amber 0.5 wt.% Cu sample, (**i**) higher magnification microscope image of the biomed amber 1.0 wt.% Cu sample, (**j**) pure biomed amber used as a control against *S. aureus*, (**k**) pure biomed amber used as a control against *E. coli*.

Figure 15 shows the results from the screening agar well diffusion test in the samples for the two bacteria tested, i.e., Gram-positive *S. aureus* and Gram-negative *E. coli*. More specifically, the developed inhibition zones (IZ) in the tests are presented. For the Gram-positive *S. aureus*, adequate width IZ was developed. As expected, the sample with the 1.0 wt.% Cu loading inhibited the Gram-positive *S. aureus* culture more intensively than the sample with 0.5 wt.% Cu loading, developing more than three times wider IZ. Results against Gram-negative *E. coli* were not as encouraging, with negligible IZ developed in both wt.% Cu loading samples. In Figure 15j the control sample is shown in the *S. aureus* culture, while Figure 15k shows the corresponding control sample in the *E. coli* culture. As expected, no inhibition zone was developed in these cases, showing that the IZs developed in the samples made with the nanocomposites were due to the addition of the Cu nanoparticles in the matrix material. Antibacterial test results are summarized in Table 7.

## 4. Discussion

The aim of this work was achieved. The properties of a commercial medical-grade resin for VPP were enriched, with the addition of Cu nanoparticles and the development of nanocomposites with low filler concentrations. This specific medical-grade resin (biomed amber, Formlabs Ohio Inc., Millbury, OH, USA) has not been investigated in the literature so far, especially for the enhancement of its mechanical properties and an attempt to induce in it antibacterial properties. The outcome of the work is not obvious by works in the literature employing different types of resins with Cu nanoparticles, as the effect of the Cu nanoparticles in the resin could differ. This was verified in the work, as the addition of the same Cu nanoparticles in the Formlabs Standard Clear V4 (Formlabs Inc., Somerville, MA, USA), improved 33.7 % the tensile strength of the resin [44], while in the current work, an improvement was achieved but with lower enhancement effect (11%). Additionally, the addition of the Cu nanoparticles induced a more effective antibacterial performance for both *S. aureus*, and *E. coli* bacteria in the Formlabs Standard Clear V4 (Formlabs Inc., Somerville, MA, USA) resin than in the biomed amber resin (Formlabs Inc., Somerville, MA, USA). If the antibacterial performance is the dominant parameter, higher Cu loading provides better results. Such materials can be employed in applications requiring enhanced mechanical performance and antibacterial behavior, further exploiting the advances of vat photopolymerization 3D printing. Such applications can be related to scaffolds and the medical or culinary field for cover parts, among others.

These nanocomposites exhibit a multifunctional performance, with enhanced mechanical properties, compared to the stock medical grade resin and biocidal behavior against two often studied in the literature bacteria. Such materials, according to the literature, are popular for use in scaffolds and devices for medical and culinary use. The low Cu concentration has two main advantages, the cytotoxicity of the materials is expected to be low [74], although no such studies were performed within the context of the work, as it was outside its purposes, and the increase in the cost from the addition of the Cu nanoparticles was negligible. The medical grade resin used cost approximately 350 €/L, which is 1090 g according to its density, so each gram cost 0.32 €. The Cu nanoparticles cost 718 €/Kg, which is 0.718 €/g. For the 0.5 wt.% loading nanocomposite the additional cost of the raw materials was 0.0036 €/g. So, the nanocomposites per gram cost 0.323 €/g, instead of the 0.32 €/g of the stock medical grade resin. Preparation costs need to be considered also, but it is not expected to be significant. These costs can be further reduced for industrial-scale use. Additionally, the thermal stability of the biomed amber was not affected by the addition of the Cu nanoparticles.

The average tensile and flexural strength of the stock biomed amber determined in the experiments agrees with the nominal values of the resin in its technical datasheet. The experimental results of the study cannot be correlated with the literature since no similar materials have been presented so far. Corresponding results with other medical grade resins as matrix materials [58,59] for the development of nanocomposites agree with the findings of the current work. The results of the work also agree with works employing Cu nanoparticles in vat photopolymerization [44,91] and material extrusion (MEX) 3D printing [92,93] for the development of nanocomposites with improved mechanical performance and antibacterial properties. The addition of Cu nanoparticles in the matrix material in these works has a similar effect to the findings of the current work.

The nanocomposites developed in the work exhibited sufficient biocidal performance against Gram-positive *S. aureus*, while the biocidal performance against Gram-negative *E. coli* was marginal, which requires further investigation. It can be attributed to the screening process followed and standardized methods should be applied for more solid conclusions regarding the antibacterial performance of the current work’s nanomaterials. In the work, minimum inhibitory concentration (MIC) analysis was not conducted, as it was outside the purposes of the work. This was not carried out since even the 0.5 wt.% concentration is very limited and lower concentrations would not be able to enable adequate mechanical reinforcement of the nanocomposites.

Apart from the antibacterial properties of the nanocomposites, the performance of the nanocomposites prepared herein was evaluated by two different categories of response parameters, i.e., parameters related to the quality characteristics of the 3D printed samples (dimensional accuracy and surface roughness) and parameters related to their mechanical performance (tensile and flexural mechanical properties). Two control parameters were used with three levels each and statistical modeling tools were employed, revealing the effect of each parameter and the relations between the parameters. These tools contributed to the analysis and optimization of the parameters studied. In the work, volumetric analysis was not conducted. Using the information from the actual to nominal deviation and the area measurements, volumetric information can be derived. This work focused on the measurements of the dimensions of the cross-section area and the analysis of any deviations, due to the 3D printing process, as the area of the samples affects the mechanical properties of the parts.

## 5. Conclusions

In this work, for the first time in the literature, a commercial medical-grade resin for VPP was reinforced with Cu nanoparticles at low concentrations, aiming also to induce antibacterial properties in the prepared nanocomposites. The aim of the work was achieved regarding the mechanical properties of the nanocomposites and partially achieved regarding the antibacterial properties. The nanocomposites inhibited the culture of the Gram-positive *S. aureus*, while they negligibly inhibited the culture of Gram-negative *E. coli* bacterium, with the process employed. The nanocomposites developed were also cost-efficient, with a multifunctional character. The nanocomposite with the 0.5 wt.% Cu loading achieved the highest mechanical performance (11% increase in the tensile strength and approximately 10% in the flexural strength), indicating that even at low concentrations the effect on the mechanical properties can be important.

The statistical analysis tool employed optimized the process and showed that both control parameters studied, i.e., wt.% Cu loading and 3D printing angle (with three levels each) have a significant effect on the two types of response parameters studied, i.e., quality parameters related to the quality of the 3D printed parts and parameters related to the mechanical properties of the materials. Following the statistical analysis, optimal dimensional accuracy is achieved with unfilled resin and 0 degrees printing angle. Optimal surface quality is achieved with unfilled resin and 90 degrees printing angle. When there is a demand for enhanced mechanical properties, nano-compounds with 0.5 wt.% Cu loading should be used, which achieved the highest mechanical response, and parts should be built with 90 degree angles.

From the regression analysis, in most cases, except for the tensile toughness (MJ/m^3^), the modeling proved reliable. Equations, providing reliable results, were prepared as functions of the control parameters, with direct industrial use for the calculation of the 11 response parameters studied, related to quality characteristics and the mechanical properties of the materials prepared in the work. According to the requirements of each application, specific response parameters can be optimized, while the others can be predicted so that the users can have an estimation of what values to anticipate. In future work, additional control parameters can be evaluated, the control parameter levels can be changed, and further studies related to the antibacterial performance of the nanocomposites prepared in this study can be conducted.

## Figures and Tables

**Table 1:** Nanocomposite elements.

#	Filler Consideration wt.% Cu	Resin
1	0.0	Biomed Amber
2	0.5	Biomed Amber
3	1.0	Biomed Amber

**Table 2:** Full factorial design: control parameters and levels.

Run	Filler Consideration wt.% Cu	Printing Angle/PA (deg.)
1	0.0	0
2	0.0	45
3	0.0	90
4	0.5	0
5	0.5	45
6	0.5	90
7	1.0	0
8	1.0	45
9	1.0	90

**Table 3:** Major Raman peaks identified and their related assignments.

Wavenumber (cm^−1^)	Raman Peak Assignment
756	O-C(O)-O stretching [109]
778	Si-O stretching [110], O-C(O)-O stretching [111]
820	phenyl ring vibration [111,112]
878	ν[O−(C=O)−O], ν(C−CH3), r(CH3), ν(ring) [109,111,113]
938	C-H bending [109,111]
1200	C-O-C stretching [109]
1243	C-O-C symmetric stretch [109]
1294	C-O-C symmetric stretch [109,111] or CH2 [112]
1364	C–C-H, C-O–H, and O-C-H [114]
1449	CH3 bending [109,111,113]
1638	v (C=C) [115]
1723	ν (C=O) [115]
2935	CH2 asymmetric stretching [114]
2953	CH2 asymmetric stretching [114]

**Table 4:** Increased Raman peaks in samples with Cu particles and their related assignments.

Wavenumber (cm^−1^)	Raman Peak Assignment	Change
756	O-C(O)-O stretching	Strong increase
778	Si-O stretching	Strong increase
820	phenyl ring vibration	Strong increase
878	ν[O−(C=O)−O], ν(C−CH3), r(CH3), ν(ring)	Strong increase
938	C-H bending	Small increase
967	Antisymmetric stretching of Si-O	A new and strong peak
1122	νs(C−C)T	A new and small peak
1200	small	Small increase
1243	small	Small increase

**Table 5:** Mean Average Values and Standard Deviations of measured Tensile Specimen Area, Tensile Specimen Area to Nominal Area, Horizontal and Vertical Ra, and Rz Roughness for each experimental run.

Run	Cross Section Area (mm^2^)	Area to Nom [%]	Ra-Hor. (μm)	Rz-Hor. (μm)	Ra-Ver. (μm)	Rz-Ver. (μm)
1	10.25 ± 0.05	100.73 ± 0.45	1.27 ± 0.07	8.46 ± 0.62	1.77 ± 0.32	12.88 ± 2.76
2	9.89 ± 0.10	97.19 ± 0.95	1.49 ± 0.04	9.82 ± 0.39	2.04 ± 0.18	12.50 ± 1.14
3	9.84 ± 0.03	96.70 ± 0.28	1.16 ± 0.21	8.00 ± 0.88	1.86 ± 0.08	11.42 ± 0.19
4	9.87 ± 0.11	96.95 ± 1.12	10.56 ± 0.59	52.20 ± 14.65	4.88 ± 0.82	28.20 ± 2.17
5	9.32 ± 0.08	91.59 ± 0.75	5.42 ± 0.39	30.60 ± 3.58	5.28 ± 0.92	25.40 ± 6.77
6	9.34 ± 0.08	91.78 ± 0.80	2.92 ± 0.36	19.20 ± 1.30	5.30 ± 1.09	27.40 ± 3.71
7	9.48 ± 0.09	93.19 ± 0.91	5.34 ± 0.42	32.00 ± 12.51	7.24 ± 0.81	33.00 ± 3.67
8	9.22 ± 0.03	90.64 ± 0.34	2.98 ± 0.58	18.80 ± 2.68	4.72 ± 0.73	29.20 ± 5.02
9	9.31 ± 0.04	91.48 ± 0.39	1.43 ± 0.20	9.74 ± 2.02	2.72 ± 0.59	15.20 ± 4.76

**Table 6:** Mean Average Values and Standard Deviations of measured Responses for Tensile Strength, Tensile Modulus of Elasticity, Tensile Toughness, Flexural Strength, Flexural Modulus of Elasticity, and Flexural Toughness for each experimental run.

Run	sB Tension (MPa)	E Tension (MPa)	Toughness Tension (MJ/m^3^)	sB Flexural (MPa)	E Flexural (MPa)	Toughness Flexural (MJ/m^3^)
1	71.91 ± 1.03	222.04 ± 20.77	20.73 ± 2.57	97.86 ± 0.59	2275.98 ± 22.19	2.68 ± 0.02
2	73.68 ± 0.58	310.31 ± 11.03	13.87 ± 0.98	104.49 ± 2.33	2616.88 ± 80.58	2.97 ± 0.08
3	71.49 ± 1.47	319.12 ± 14.97	10.97 ± 2.13	108.06 ± 1.79	2746.75 ± 63.12	3.10 ± 0.06
4	78.63 ± 1.96	320.85 ± 16.06	14.97 ± 1.24	109.42 ± 1.52	2573.82 ± 38.04	3.05 ± 0.04
5	80.96 ± 0.74	345.99 ± 12.10	15.53 ± 1.40	113.49 ± 2.48	2811.18 ± 68.65	3.26 ± 0.07
6	82.53 ± 0.24	350.18 ± 10.44	16.69 ± 1.11	115.37 ± 2.63	2911.61 ± 75.71	3.40 ± 0.09
7	78.20 ± 1.14	297.76 ± 14.04	15.84 ± 0.30	101.27 ± 0.80	2350.87 ± 24.04	2.81 ± 0.03
8	75.53 ± 2.73	330.86 ± 7.98	15.56 ± 6.19	109.33 ± 2.51	2710.62 ± 60.43	3.14 ± 0.06
9	80.04 ± 0.58	343.17 ± 2.53	14.97 ± 1.78	114.03 ± 1.11	2892.06 ± 73.77	3.30 ± 0.06

**Table 7:** Antibacterial test results.

Nano Compound wt.% Cu	*S. aureus* IZ (mm)	*E. coli* IZ (mm)
0.0	0.0	0.0
0.5	2.0	~0.5
1.0	7.0	~0.5

## Data Availability

The data presented in this study are available upon request from the corresponding author.

## Supplementary Materials

The following supporting information can be downloaded at: https://www.mdpi.com/article/10.3390/jfb13040258/s1, Figure S1. Pareto chart and experimental vs. calculated (predicted) graph, for (a) tensile strength (MPa), (b) tensile modulus of elasticity (GPa), (c) tensile toughness (MJ/m^3^), Figure S2. Pareto chart and experimental vs. calculated (predicted) graph, for (a) flexural strength (MPa), (b) flexural modulus of elasticity (GPa), (c) flexural toughness (MJ/m^3^), Figure S3. Pareto chart and experimental vs. calculated (predicted) graph, for (a) cross-section area to nominal (%), (b) cross-section area (mm^2^), Figure S4. Pareto chart and experimental vs. calculated (predicted) graph, for (a) surface roughness Ra in the horizontal direction (μm) (b) surface roughness Ra in the vertical direction (μm), (c) surface roughness Rz in the horizontal direction (μm) (d) surface roughness Rz in the vertical direction (μm), Table S1. Measured Tensile Strength, Tensile Modulus of Elasticity, Tensile Toughness, Flexural Strength, Flexural Modulus of Elasticity, and Flexural Toughness for each experimental run and five replicas per run, Table S2. Measured Tensile Specimen Area, Tensile Specimen Area to Nominal Area, Horizontal and Vertical Ra and Rz Roughness for each experimental run and five replicas per run, Table S3. Polynomial ANOVA, Tensile Strength vs. %Cu, PA., Table S4. Polynomial ANOVA, Tensile Modulus of Elasticity vs. %Cu, PA., Table S5. Polynomial ANOVA, Tensile Toughness vs. %Cu, PA., Table S6. Polynomial ANOVA, Flexural Strength vs. %Cu, PA., Table S7. Polynomial ANOVA, Flexural Modulus of Elasticity vs. %Cu, PA., Table S8. Polynomial ANOVA, Flexural Toughness vs. %Cu, PA., Table S9. Polynomial ANOVA, Measured area of Specimen, PA., Table S10. Polynomial ANOVA, Measured Area to Nominal vs. %Cu, PA., Table S11. Polynomial ANOVA, Ra Roughness—Horizontal vs. %Cu, PA., Table S12. Polynomial ANOVA, Rz Roughness—Horizontal vs. %Cu, PA., Table S13. Polynomial ANOVA, Ra Roughness—Vertical vs. %Cu, PA., Table S14. Polynomial ANOVA, Rz Roughness—Vertical vs. %Cu, PA.
