# Supplementary material for: Multifunctional Medical Grade Resin with Enhanced Mechanical and Antibacterial Properties: The Effect of Copper Nano-Inclusions in Vat Polymerization (VPP) Additive Manufacturing
Source: J Funct Biomater. 2022 Nov 21;13(4):258. doi: 10.3390/jfb13040258 (PMC9680439; doi:10.3390/jfb13040258)
Supplement: Supplementary file 1 [file jfb-13-00258-s001.zip › jfb-2030620-supplementary.pdf]

Journal of *Functional Biomaterials*

MDPI

Supplementary material for the Article

Multifunctional Medical Grade Resin with enhanced mechanical and antibacterial properties: The effect of Copper Nano-Inclusions in VAT polymerization Additive Manufacturing

N. Vidakis [1*], M. Petousis [1], V. Papadakis [2], N. Mountakis [1]

[1] Department of Mechanical Engineering, Hellenic Mediterranean University, Heraklion 71410, Greece, vidakis@hmu.gr (N.V.); markospetousis@hmu.gr (M.P.); mountakis@hmu.gr (N.M.)
[2] Institute of Molecular Biology and Biotechnology, Foundation for Research and Technology – Hellas, 71110 Heraklion, Greece., vassilis_papadakis@imbb.forth.gr (V.P.)
* Corresponding author: e-mail: vidakis@hmu.gr (N.V.), Tel.: +302810379227)

1. Experimental results

The complete experimental results for the control parameters' different levels are presented in tables S1 and S2.

Table S1. Measured Tensile Strength, Tensile Modulus of Elasticity, Tensile Toughness, Flexural Strength, Flexural Modulus of Elasticity, and Flexural Toughness for each experimental run and five replicas per run.

A/A	%Cu (%)	Printing Angle	sB Tension (MPa)	E Tension (MPa)	Toughness Tension	sB Flexural (MPa)	E Flexural (MPa)	Toughness Flexural (MJ/m³)
1	0.0	0	73.71	194.48	21.28	98.59	2308.27	2.71
2	0.0	0	71.26	242.17	16.19	97.14	2247.49	2.65
3	0.0	0	71.78	232.20	21.85	97.47	2282.37	2.68
4	0.0	0	71.50	205.62	22.49	97.78	2274.25	2.67
5	0.0	0	71.27	235.74	21.84	98.31	2267.52	2.67
6	0.0	45	73.38	294.86	15.29	106.07	2642.36	3.03
7	0.0	45	74.62	320.78	14.18	101.21	2505.51	2.86
8	0.0	45	73.33	311.37	12.59	107.20	2682.39	3.05
9	0.0	45	73.25	304.24	13.57	103.41	2562.18	2.95
10	0.0	45	73.85	320.32	13.74	104.58	2691.97	2.97
11	0.0	90	69.15	317.60	8.43	108.66	2779.74	3.13
12	0.0	90	72.25	294.25	13.26	109.35	2797.50	3.15
13	0.0	90	72.71	330.93	12.04	104.93	2637.71	3.00
14	0.0	90	72.38	330.22	12.14	108.33	2765.07	3.13
15	0.0	90	70.96	322.63	8.98	109.01	2753.75	3.11
16	0.5	0	81.70	341.88	17.05	108.21	2561.64	3.03
17	0.5	0	77.23	311.29	14.51	111.52	2640.81	3.11
18	0.5	0	78.11	312.32	14.00	108.77	2552.40	3.03

19	0.5	0	76.82	333.86	14.17	110.52	2548.86	3.03
20	0.5	0	79.30	304.87	15.13	108.08	2565.40	3.06
21	0.5	45	81.08	336.08	16.97	109.98	2730.28	3.15
22	0.5	45	79.86	342.05	13.69	114.25	2826.24	3.27
23	0.5	45	81.80	361.07	16.56	116.05	2877.97	3.32
24	0.5	45	80.67	356.43	14.48	115.20	2748.97	3.32
25	0.5	45	81.40	334.33	15.97	111.96	2872.42	3.22
26	0.5	90	82.32	366.04	16.94	118.73	2994.90	3.44
27	0.5	90	82.39	342.41	15.45	112.30	2825.93	3.25
28	0.5	90	82.84	346.34	18.23	117.40	2987.58	3.40
29	0.5	90	82.36	355.15	15.78	114.53	2884.08	3.46
30	0.5	90	82.76	340.96	17.02	113.87	2865.55	3.45
31	1.0	0	76.34	317.43	15.99	102.05	2370.41	2.82
32	1.0	0	78.07	282.18	16.27	100.39	2322.19	2.77
33	1.0	0	79.32	304.43	15.56	102.17	2380.58	2.83
34	1.0	0	78.80	287.05	15.58	100.99	2343.17	2.80
35	1.0	0	78.49	297.70	15.80	100.73	2338.01	2.83
36	1.0	45	75.19	341.44	8.98	110.21	2695.02	3.15
37	1.0	45	79.03	320.40	21.40	106.22	2628.71	3.04
38	1.0	45	71.81	329.24	9.00	111.82	2772.77	3.20
39	1.0	45	74.51	327.75	20.96	111.28	2688.51	3.17
40	1.0	45	77.13	335.48	17.46	107.15	2768.08	3.15
41	1.0	90	79.32	342.01	12.87	112.47	2796.76	3.22
42	1.0	90	79.97	340.48	17.07	114.15	2861.32	3.28
43	1.0	90	80.67	346.04	16.42	115.14	2993.17	3.37
44	1.0	90	79.64	341.61	13.65	114.95	2929.09	3.28
45	1.0	90	80.58	345.69	14.86	113.43	2879.98	3.35
		Min	69.15	194.48	8.43	97.14	2247.49	2.65
		Max	82.84	366.04	22.49	118.73	2994.90	3.46
		Average	77.00	315.59	15.46	108.15	2654.42	3.08

Table S2. Measured Tensile Specimen Area, Tensile Specimen Area to Nominal Area, Horizontal and Vertical Ra and Rz Roughness for each experimental run and five replicas per run.

**A/A**	**%Cu (%)**	**Printing Angle (deg.)**	**Area Tension**	**Area to Nom [%]**	**Ra-Hor. (μm)**	**Rz-Hor. (μm)**	**Ra-Ver. (μm)**	**Rz-Ver. (μm)**
1	0.0	0	10.31	101.27	1.18	7.8	1.78	11.1
2	0.0	0	10.28	100.97	1.24	8.1	2.30	15.6
3	0.0	0	10.18	100.05	1.34	9.4	1.46	9.0

4	0.0	0	10.24	100.68	1.33	8.3	1.63	13.8
5	0.0	0	10.24	100.68	1.28	8.7	1.68	14.9
6	0.0	45	9.77	95.96	1.48	9.2	2.02	12.2
7	0.0	45	9.92	97.50	1.54	9.7	2.16	13.8
8	0.0	45	10.01	98.41	1.46	10.2	1.74	11.4
9	0.0	45	9.83	96.58	1.52	10.0	2.18	13.6
10	0.0	45	9.92	97.50	1.46	10.0	2.08	11.5
11	0.0	90	9.86	96.89	1.18	7.3	1.82	11.3
12	0.0	90	9.86	96.89	0.98	6.9	1.78	11.7
13	0.0	90	9.80	96.27	1.50	9.0	1.98	11.2
14	0.0	90	9.86	96.89	0.99	8.6	1.86	11.4
15	0.0	90	9.83	96.58	1.15	8.2	1.84	11.5
16	0.5	0	9.94	97.67	9.70	63.0	5.80	29.0
17	0.5	0	9.97	98.01	11.00	33.0	4.60	27.0
18	0.5	0	9.69	95.22	11.00	65.0	4.00	25.0
19	0.5	0	9.91	97.39	10.90	40.0	5.70	30.0
20	0.5	0	9.82	96.45	10.20	60.0	4.30	30.0
21	0.5	45	9.36	92.01	5.00	31.0	5.60	23.0
22	0.5	45	9.36	92.01	5.80	36.0	4.40	28.0
23	0.5	45	9.21	90.52	5.00	26.0	6.60	36.0
24	0.5	45	9.27	91.12	5.70	30.0	5.40	20.0
25	0.5	45	9.39	92.31	5.60	30.0	4.40	20.0
26	0.5	90	9.39	92.32	2.60	17.0	4.20	21.0
27	0.5	90	9.39	92.32	3.40	19.0	4.40	28.0
28	0.5	90	9.21	90.52	2.60	20.0	5.00	30.0
29	0.5	90	9.30	91.42	2.80	20.0	6.40	30.0
30	0.5	90	9.39	92.32	3.20	20.0	6.50	28.0
31	1.0	0	9.39	92.26	5.30	53.0	8.20	33.0
32	1.0	0	9.60	94.30	5.00	26.0	6.80	39.0
33	1.0	0	9.45	92.83	5.60	31.0	6.20	33.0
34	1.0	0	9.57	94.00	4.90	30.0	7.10	30.0
35	1.0	0	9.42	92.54	5.90	20.0	7.90	30.0
36	1.0	45	9.21	90.52	3.40	20.0	3.60	22.0
37	1.0	45	9.18	90.22	2.00	14.0	5.40	36.0
38	1.0	45	9.24	90.82	3.00	20.0	5.00	28.0
39	1.0	45	9.21	90.52	3.40	20.0	4.40	30.0
40	1.0	45	9.27	91.12	3.10	20.0	5.20	30.0
41	1.0	90	9.33	91.72	1.24	9.1	3.60	15.0
42	1.0	90	9.24	90.82	1.36	6.6	2.80	20.0
43	1.0	90	9.33	91.72	1.68	11.9	2.00	11.0
44	1.0	90	9.30	91.42	1.61	10.8	2.80	10.0
45	1.0	90	9.33	91.72	1.26	10.3	2.40	20.0

Min	9.18	90.22	0.98	6.6	1.46	9.0
Max	10.31	101.27	11.00	65.0	8.20	39.0
Average	9.61	94.47	3.62	21.0	3.98	21.7

2. ANOVA analysis tables

The following tables present the ANOVA analysis for the response vs. the control parameters.

Table S3. Polynomial ANOVA, Tensile Strength vs %Cu, PA.

Source	DF	Adj SS	Adj MS	F-Value	P-Value
Regression	5	573.872	114.774	31.96	0.000
%Cu	1	369.069	369.069	102.78	0.000
PA	1	0.497	0.497	0.14	0.712
$\%Cu^2$	1	310.023	310.023	86.34	0.000
PA^2	1	1.659	1.659	0.46	0.501
%Cu × PA	1	6.327	6.327	1.76	0.192
Error	39	140.046	3.591		
Total	44	713.918	118.365		
R^2	80.38%				
R^2 (adj)	77.87%				
R^2 (pred)	74.24%				

Table S4. Polynomial ANOVA, Tensile Modulus of Elasticity vs %Cu, PA.

Source	DF	Adj SS	Adj MS	F-Value	P-Value
Regression	5	56424	11284.7	39.78	0.000
%Cu	1	22046	22045.6	77.71	0.000
PA	1	13899	13899.3	48.99	0.000
$\%Cu^2$	1	12340	12339.9	43.50	0.000
PA^2	1	4082	4082.1	14.39	0.001
%Cu × PA	1	3337	3337.3	11.76	0.001
Error	39	11064	283.7		
Total	44	67488	11568.4		
R^2	83.61%				
R^2 (adj)	81.50%				
R^2 (pred)	78.11%				

Table S5. Polynomial ANOVA, Tensile Toughness vs %Cu, PA.

Source	DF	Adj SS	Adj MS	F-Value	P-Value
Regression	5	172.317	34.463	4.14	0.004
%Cu	1	3.380	3.380	0.41	0.528

PA	1	54.246	54.246	6.52	0.015
%Cu^2	1	1.651	1.651	0.20	0.658
PA^2	1	4.974	4.974	0.60	0.444
%Cu × PA	1	98.933	98.933	11.89	0.001
Error	39	324.426	8.319		
Total	44	496.743	42.782		
R^2	34.69%				
R^2 (adj)	26.32%				
R^2 (pred)	14.60%				

Table S6. Polynomial ANOVA, Flexural Strength vs %Cu, PA.

Source	DF	Adj SS	Adj MS	F-Value	P-Value
Regression	5	1372.42	274.484	58.81	0.000
%Cu	1	501.27	501.267	107.40	0.000
PA	1	103.00	103.001	22.07	0.000
%Cu^2	1	478.61	478.605	102.54	0.000
PA^2	1	20.72	20.715	4.44	0.042
%Cu × PA	1	8.21	8.212	1.76	0.192
Error	39	182.03	4.667		
Total	44	1554.45	279.151		
R^2	88.29%				
R^2 (adj)	86.79%				
R^2 (pred)	84.79%				

Table S7. Polynomial ANOVA, Flexural Modulus of Elasticity vs %Cu, PA.

Source	DF	Adj SS	Adj MS	F-Value	P-Value
Regression	5	1961254	392251	85.18	0.000
%Cu	1	280289	280289	60.87	0.000
PA	1	303135	303135	65.83	0.000
%Cu^2	1	277802	277802	60.33	0.000
PA^2	1	76929	76929	16.71	0.000
%Cu × PA	1	6198	6198	1.35	0.253
Error	39	179586	4605		
Total	44	2140840	396856		
R^2	91.61%				
R^2 (adj)	90.54%				
R^2 (pred)	88.96%				

Table S8. Polynomial ANOVA, Flexural Toughness vs %Cu, PA.

Source	DF	Adj SS	Adj MS	F-Value	P-Value
Regression	5	2.14491	0.428982	107.40	0.000
%Cu	1	0.59972	0.599721	150.14	0.000
PA	1	0.22027	0.220266	55.14	0.000
$\%Cu^2$	1	0.55425	0.554255	138.76	0.000
PA^2	1	0.04323	0.043227	10.82	0.002
%Cu × PA	1	0.00512	0.005119	1.28	0.265
Error	39	0.15578	0.003994		
Total	44	2.30069	0.432976		
R^2	93.23%				
R^2 (adj)	92.36%				
R^2 (pred)	91.16%				

Table S9. Polynomial ANOVA, Measured area of Specimen, PA.

Source	DF	Adj SS	Adj MS	F-Value	P-Value
Regression	5	2.14491	0.428982	107.40	0.000
%Cu	1	0.59972	0.599721	150.14	0.000
PA	1	0.22027	0.220266	55.14	0.000
$\%Cu^2$	1	0.55425	0.554255	138.76	0.000
PA^2	1	0.04323	0.043227	10.82	0.002
%Cu × PA	1	0.00512	0.005119	1.28	0.265
Error	39	0.15578	0.003994		
Total	44	2.30069	0.432976		
R^2	93.97%				
R^2 (adj)	93.20%				
R^2 (pred)	92.12%				

Table S10. Polynomial ANOVA, Measured Area to Nominal vs %Cu, PA.

Source	DF	Adj SS	Adj MS	F-Value	P-Value
Regression	5	480.815	96.1630	121.59	0.000
%Cu	1	98.358	98.3583	124.37	0.000
PA	1	84.430	84.4300	106.76	0.000
$\%Cu^2$	1	23.956	23.9556	30.29	0.000
PA^2	1	39.835	39.8351	50.37	0.000
%Cu × PA	1	6.696	6.6959	8.47	0.006
Error	39	30.843	0.7909		
Total	44	511.658	96.9539		
R^2	93.97%				
R^2 (adj)	93.20%				

R^2 (pred)	92.12%

Table S11. Polynomial ANOVA, Ra Roughness - Horizontal vs %Cu, PA.

Source	DF	Adj SS	Adj MS	F-Value	P-Value
Regression	5	323.646	64.729	40.75	0.000
%Cu	1	205.286	205.286	129.23	0.000
PA	1	7.962	7.962	5.01	0.031
$\%Cu^2$	1	161.658	161.658	101.76	0.000
PA^2	1	2.336	2.336	1.47	0.233
%Cu × PA	1	18.012	18.012	11.34	0.002
Error	39	61.954	1.589		
Total	44	385.600	66.318		
R^2	83.93%				
R^2 (adj)	81.87%				
R^2 (pred)	78.89%				

Table S12. Polynomial ANOVA, Rz Roughness - Horizontal vs %Cu, PA.

Source	DF	Adj SS	Adj MS	F-Value	P-Value
Regression	5	8008.24	1601.65	25.51	0.000
%Cu	1	5218.03	5218.03	83.11	0.000
PA	1	118.14	118.14	1.88	0.178
$\%Cu^2$	1	3814.21	3814.21	60.75	0.000
PA^2	1	34.60	34.60	0.55	0.462
%Cu × PA	1	594.05	594.05	9.46	0.004
Error	39	2448.51	62.78		
Total	44	10456.75	1664.43		
R^2	76.58%				
R^2 (adj)	73.58%				
R^2 (pred)	69.00%				

Table S13. Polynomial ANOVA, Ra Roughness - Vertical vs %Cu, PA.

Source	DF	Adj SS	Adj MS	F-Value	P-Value
Regression	5	138.824	27.7648	36.47	0.000
%Cu	1	79.032	79.0315	103.81	0.000
PA	1	0.707	0.7068	0.93	0.341
$\%Cu^2$	1	31.082	31.0817	40.82	0.000
PA^2	1	0.026	0.0260	0.03	0.854
%Cu × PA	1	26.519	26.5190	34.83	0.000
Error	39	29.692	0.7613		
Total	44	168.516	28.5261		

R^2	82.38%
R^2 (adj)	80.12%
R^2 (pred)	76.92%

Table S14. Polynomial ANOVA, Rz Roughness - Vertical vs %Cu, PA.

Source	DF	Adj SS	Adj MS	F-Value	P-Value
Regression	5	2687.73	537.55	27.42	0.000
%Cu	1	1484.35	1484.35	75.71	0.000
PA	1	15.93	15.93	0.81	0.373
$\%Cu^2$	1	634.68	634.68	32.37	0.000
PA^2	1	10.34	10.34	0.53	0.472
%Cu × PA	1	333.74	333.74	17.02	0.000
Error	39	764.62	19.61		
Total	44	3452.35	557.16		
R^2	77.85%				
R^2 (adj)	75.01%				
R^2 (pred)	70.90%				

3. Pareto charts for the response vs the control parameters

The following figures present the Pareto charts for the response vs. the control parameters and a comparison between the experimental and the calculated with the produced model values.

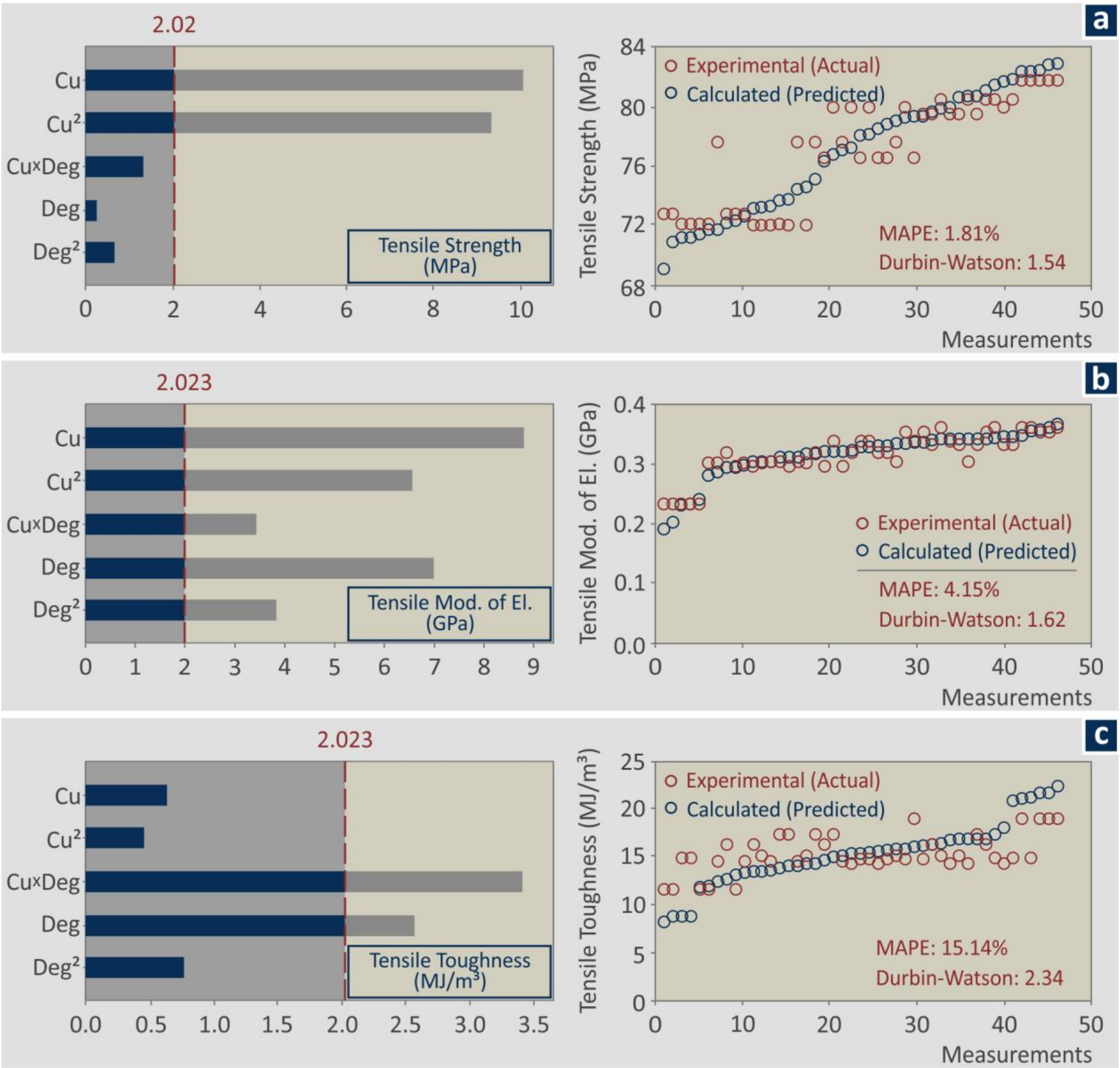

Figure S1. Pareto chart and experimental vs calculated (predicted) graph, for (a) tensile strength (MPa), (b) tensile modulus of elasticity (GPa), (c) tensile toughness (MJ/m^3)

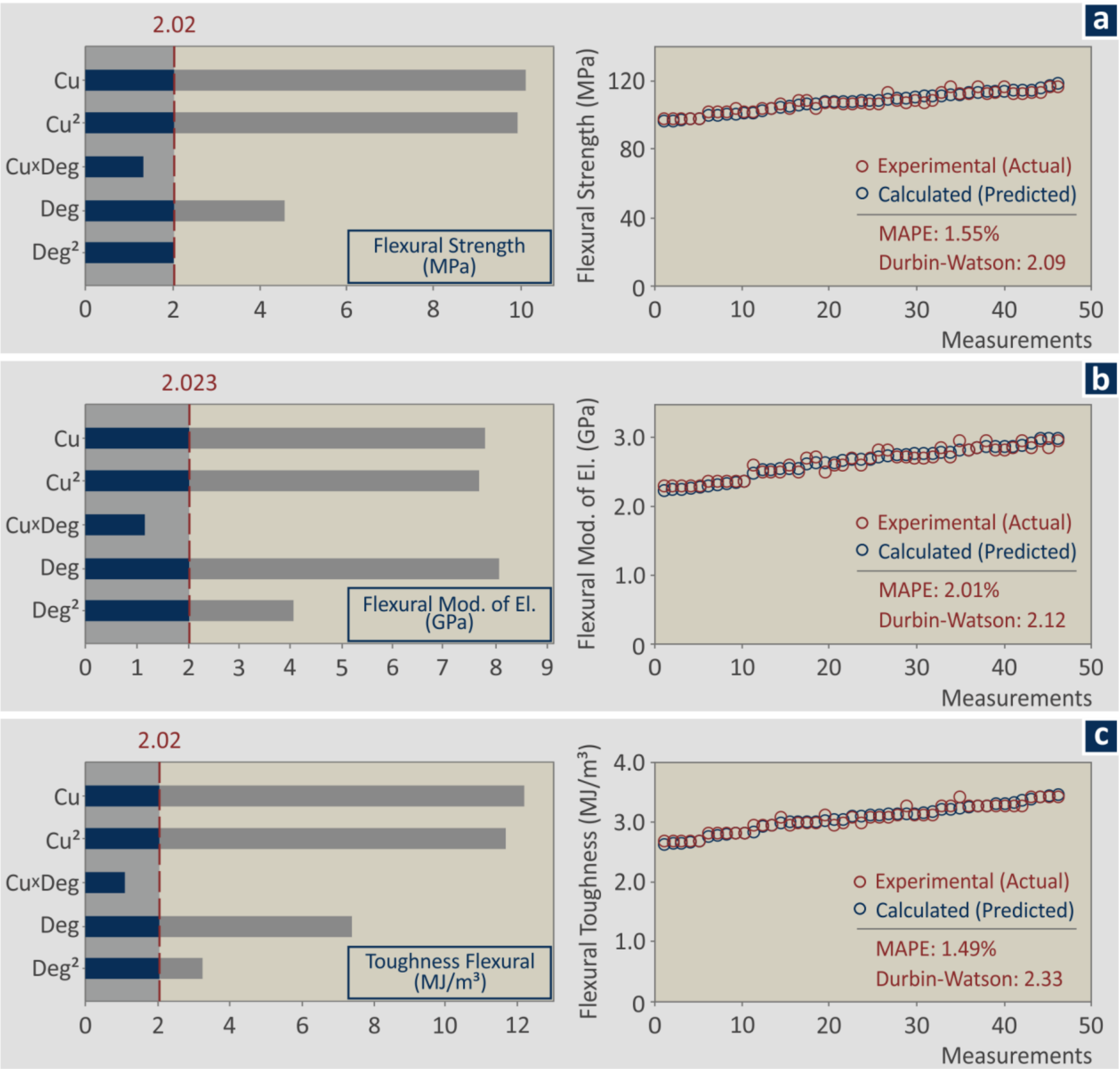

Figure S2. Pareto chart and experimental vs calculated (predicted) graph, for (a) flexural strength (MPa), (b) flexural modulus of elasticity (GPa), (c) flexural toughness (MJ/m^3)

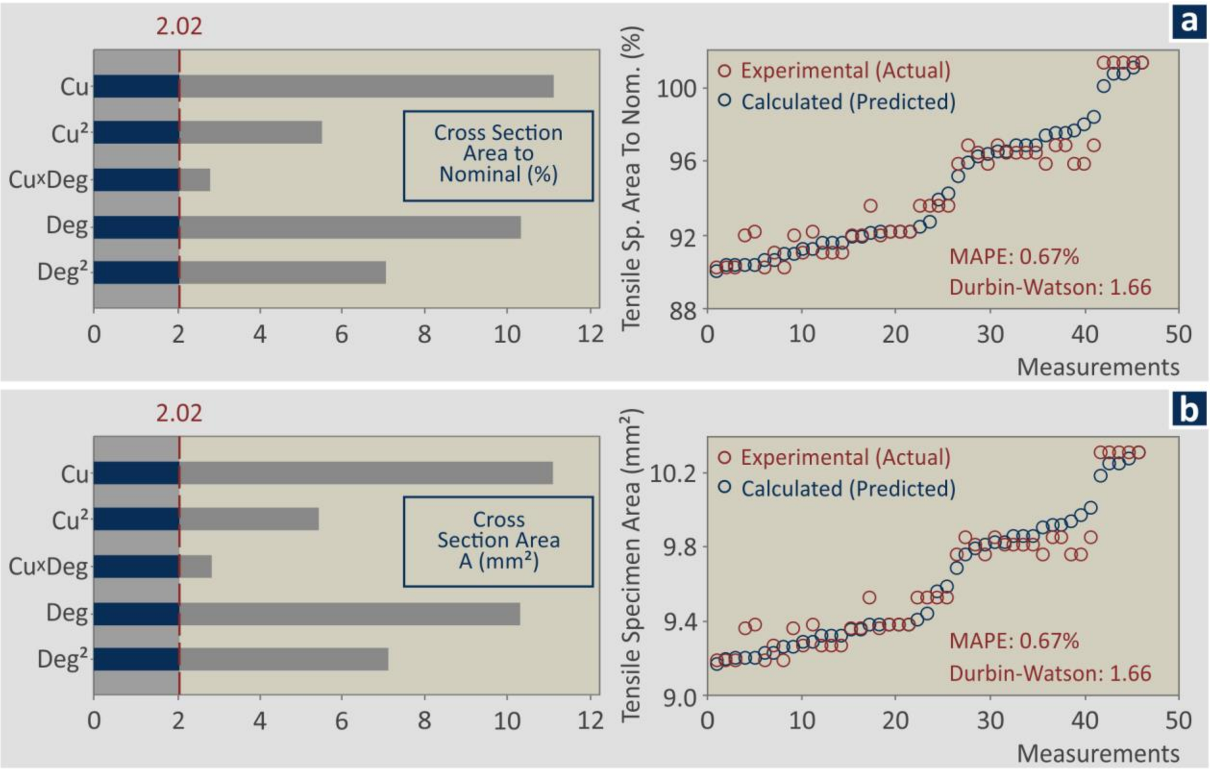

Figure S3. Pareto chart and experimental vs calculated (predicted) graph, for (a) cross-section area to nominal (%), (b) cross-section area (mm^2)

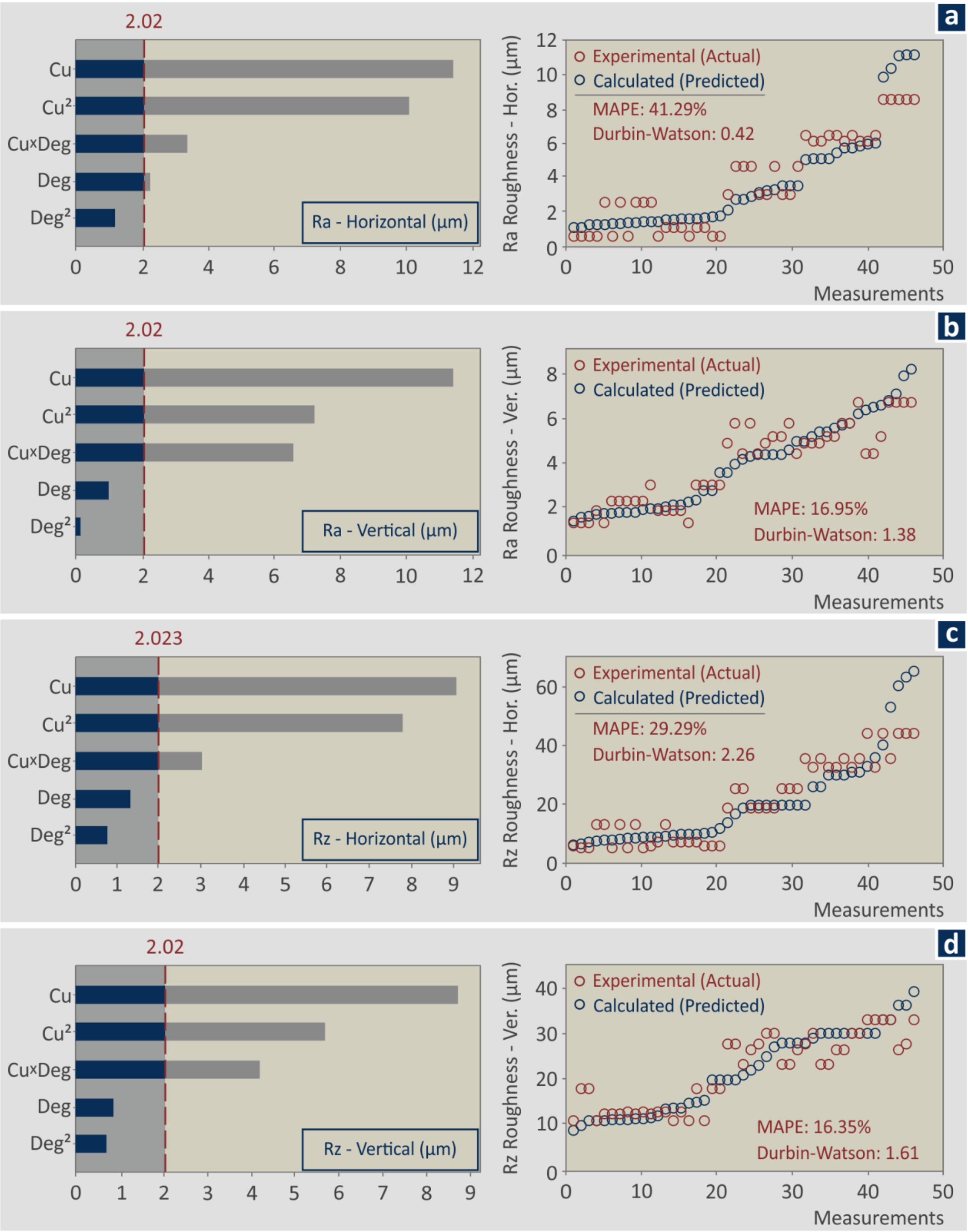

Figure S4. Pareto chart and experimental vs calculated (predicted) graph, for (a) surface roughness Ra in the horizontal direction (µm) (b) surface roughness Ra in the vertical direction (µm), (c) surface roughness Rz in the horizontal direction (µm) (d) surface roughness Rz in the vertical direction (µm).
